# Mapping study for health emergency and disaster risk management competencies and curricula: literature review and cross-sectional survey

**DOI:** 10.1186/s12992-023-01010-y

**Published:** 2024-02-21

**Authors:** Kevin K. C. Hung, Makiko K. MacDermot, Theresa S. I. Hui, Suet Yi Chan, Sonoe Mashino, Catherine P. Y. Mok, Pak Ho Leung, Ryoma Kayano, Jonathan Abrahams, Chi Shing Wong, Emily Y. Y. Chan, Colin A. Graham

**Affiliations:** 1 Accident and Emergency Medicine Academic Unit, The Chinese University of Hong Kong, Trauma & Emergency Centre, Prince of Wales Hospital, Shatin, New Territories Hong Kong; 2 Collaborating Centre for Oxford University and Chinese University of Hong Kong for Disaster and Medical Humanitarian Response (CCOUC), JC School of Public Health and Primary Care, The Chinese University of Hong Kong, Hong Kong, China; 3 Research Institute of Nursing Care for People and Community, University of Hyogo, Akashi, 673-8588 Japan; 4 World Health Organization Centre for Health Development, Kobe, 651-0073 Japan; 5 Monash University Disaster Resilience Initiative, Monash University, Clayton, Australia

**Keywords:** Health emergency and disaster risk management, Competency model, Curriculum, Health workforce

## Abstract

**Background:**

With the increasing threat of hazardous events at local, national, and global levels, an effective workforce for health emergency and disaster risk management (Health EDRM) in local, national, and international communities is urgently needed. However, there are no universally accepted competencies and curricula for Health EDRM. This study aimed to identify Health EDRM competencies and curricula worldwide using literature reviews and a cross-sectional survey.

**Methods:**

Literature reviews in English and Japanese languages were performed. We searched MEDLINE, EMBASE, CINAHL (English), and the ICHUSHI (Japanese) databases for journal articles published between 1990 and 2020. Subsequently, a cross-sectional survey was sent to WHO Health EDRM Research Network members and other recommended experts in October 2021 to identify competency models and curricula not specified in the literature search.

**Results:**

Nineteen studies from the searches were found to be relevant to Health EDRM competencies and curricula. Most of the competency models and curricula were from the US. The domains included knowledge and skills, emergency response systems (including incident management principles), communications, critical thinking, ethical and legal aspects, and managerial and leadership skills. The cross-sectional survey received 65 responses with an estimated response rate of 25%. Twenty-one competency models and 20 curricula for managers and frontline personnel were analyzed; managers' decision-making and leadership skills were considered essential.

**Conclusion:**

An increased focus on decision-making and leadership skills should be included in Health EDRM competencies and curricula to strengthen the health workforce.

**Supplementary Information:**

The online version contains supplementary material available at 10.1186/s12992-023-01010-y.

## Introduction

Disasters have been defined by the World Health Organization (WHO) as “serious disruptions of the functioning of a community or a society at any scale due to hazardous events interacting with conditions of exposure, vulnerability and capacity, leading to one or more of the following: human, material, economic and environmental losses and impacts” [1]. The consequences of disasters are often devastating, leading to high mortality and imposing extreme burdens on health systems and national economies [2–7]. During the 2010s, an average of approximately 45,000 people died annually from disasters associated with natural hazards globally, around 0.1% of total deaths [2].

The latest coronavirus disease (COVID-19) pandemic demonstrated how biological hazards could overwhelm health systems in both developing and developed countries. As of March 2023, over 758 million people had been infected globally, resulting in more than 6.8 million deaths [4].

With the increasing threat of hazardous events at local, national, and global levels, an effective workforce for health emergency and disaster risk management (Health EDRM) in local, national, and international communities is urgently needed [8]. Health EDRM emphasizes assessing, communicating, and reducing risks across all phases of the disaster cycle and building the resilience of communities, countries, and health systems. Health EDRM requires collective action by health systems, communities, and partners across society to reduce health risks and the consequences of all types of emergencies and disasters [8].

Enhancing health workforce capacity and competencies has long been an international goal in disaster risk reduction. According to the 2015 Sendai Framework for Disaster Risk Reduction, strengthening the training capacities in disaster medicine and training the healthcare workforce in disaster risk reduction are important ways to achieve effective disaster risk reduction and enhance resilience [9]. The WHO recognizes that a skilled and trained health workforce is imperative for countries to effectively implement disaster risk management, including emergency preparedness measures [10]. In the WHO Global Strategy on Human Resources for Health 2030, technical support to health system capacities and workforce competency is considered one of the Secretariat's core activities [10].

Studies have shown that receiving training and having relevant knowledge and skills for roles in emergencies and disasters are significantly related to increased willingness to work in an emergency and higher confidence in performing duties or functions in disaster situations [11, 12]. The global competency framework for universal health coverage has also included “developing preparedness for health emergencies and disasters, including disease outbreaks” and “responding to health emergencies and disasters, including disease outbreaks” in the list of core functions of health practice, which require relevant training in Health EDRM core competencies [13].

While the importance of disaster health education and training is well recognized, there is a need for an evidence-based approach to the training contents and their delivery for the wide range of roles in the health workforce. Defining the core competencies in knowledge and skills in Health EDRM for this health workforce is challenging. Previous literature reviews failed to identify an agreed set of competencies for disaster healthcare providers [14–18]. There were also wide variations in training delivery modalities and evaluation methods. However, for chemical, biological, radiological, and nuclear (CBRN) incident response, scenario-based training appeared to be more effective than other types of training [18, 19]. Furthermore, there has been insufficient research on the long-term impact of emergency preparedness training and exercises on individuals and their effectiveness in actual events [18, 20]. As shown in a review of training for the WHO Ebola emergency response, most published articles focused on competencies for a single profession instead of adopting a multidisciplinary approach [21]. Building standardized and accredited core competencies for all healthcare workers in Health EDRM is necessary to ensure the delivery of safe and quality care in disasters.

As a first step in the health workforce Health EDRM capacity building effort, the WHO Health Emergencies Programme (WHE) Learning Strategy was developed in 2018 to provide standards and frameworks in training. This strategy is intended for all WHO Health Emergency personnel, WHO partners, and volunteers at national, regional, and international levels [22]. It introduced the WHE Competency Framework based on the Competency = Attributes + Skills + Knowledge (CASK) model with attributes, skills, and knowledge as key components of competencies. The WHE Training Framework was also established to train WHO staff at different levels through online courses, face-to-face training, and simulation exercises [22]. However, the applicability of the competency and training frameworks is still being piloted at the time of writing. Further evidence for the essential elements of WHE competencies and their ideal training modalities will be crucial before their wider dissemination.

The WHO Health EDRM Research Network (Health EDRM RN) has previously reported the need to identify knowledge and evidence gaps in the capacity development of the health workforce with respect to Health EDRM [23, 24].

The present study aimed to address the evidence gap by identifying the competencies and curricula currently used by disaster risk management-related agencies. It alsoexplored the knowledge gap of the match between these existing competencies and curricula on the one hand, and the WHE core Competency and Training Frameworks and principles of Health EDRM on the other hand. Our research question was therefore: do the current competency models and curricula match the WHE core competency domains and the comprehensive emergency management and risk-based approach of Health EDRM?

## Materials and methods

This competency mapping study was part of a research project funded by the WHO Centre for Health Development (WHO Kobe Centre - WKC) under the Health Workforce Development for Health EDRM (Research Area 4 of WKC’s four key research themes). This competency mapping study included a literature review and a cross-sectional survey, the latter of which was included because some competency models and curricula may have been missed in the literature review. The literature review was performed in the English and Japanese languages using a systematic approach to the literature search. Searches of the English language literature published from 1 January 1990 to 11 March 2020 were conducted using MEDLINE (1966), EMBASE (1980), and CINAHL (1980). The Japanese language literature search was performed using the ICHUSHI database and included articles published from 1 January 1990 to 23 October 2020. The English and Japanese search terms can be found in the Supplementary file 1.

For both languages, we included only papers matching the definition of disaster or humanitarian crisis and those relevant to the competencies and curricula of Health EDRM. We excluded papers based purely on military settings or on training for a single type of clinical procedure or surgery, papers focusing on research methodologies, as well as conference abstracts and papers where full-text versions were not available at the university library systems in Hong Kong or Japan.

**Fig. 1:**
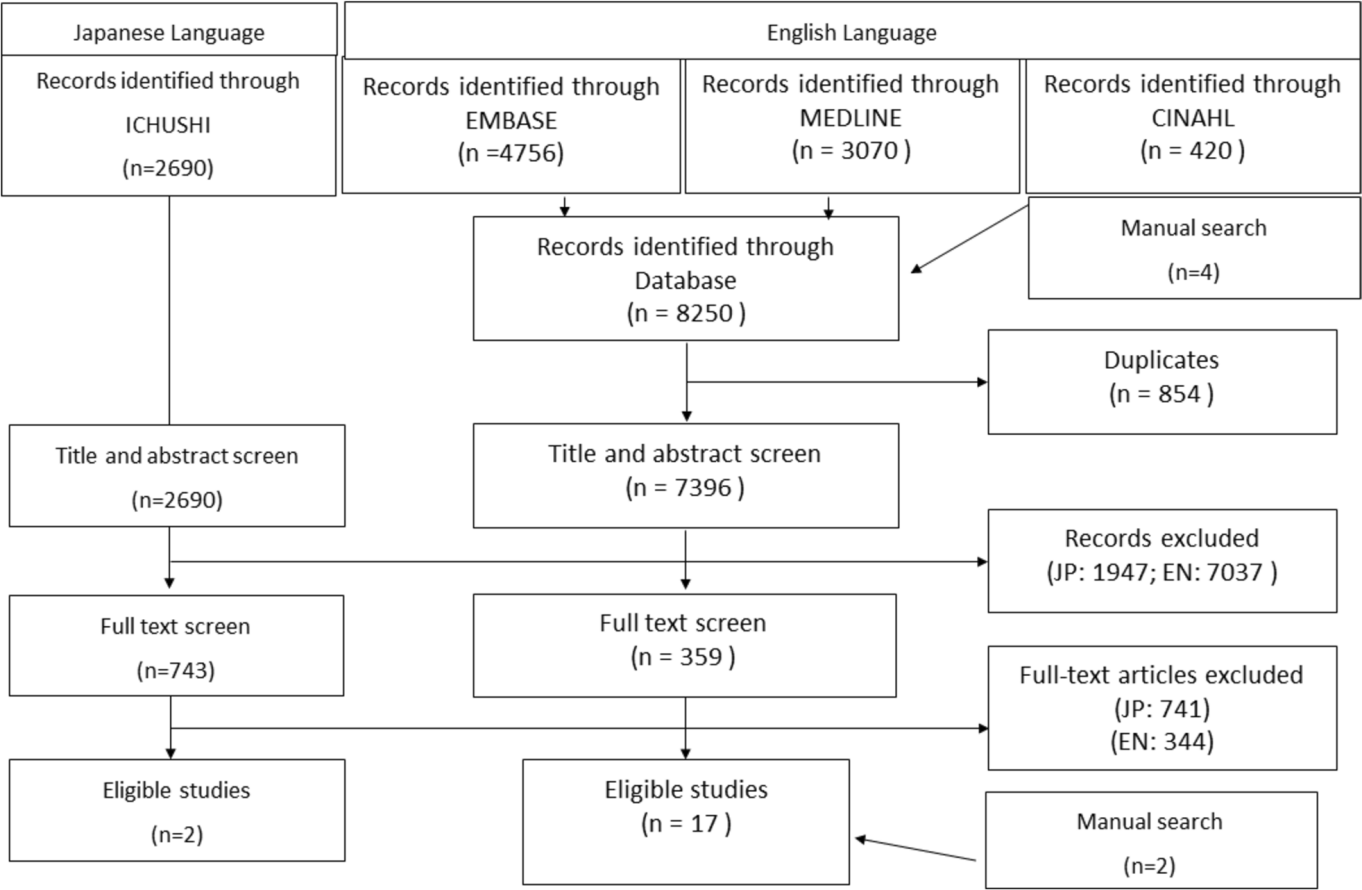
PRISMA flow chart

Title and abstract screening were performed by a single reviewer for the 7,396 English-language and the 2,690 Japanese-language articles. A total of 359 English-language and 743 Japanese-language articles were selected and underwent full-text screening by two reviewers. If disagreement between the two reviewers about the suitability of an article could not be resolved after discussion, a third reviewer was invited to review the article independently. After this procedure, two more competency models were identified from a subsequent manual search. Finally, seventeen eligible articles in English language and two articles in Japanese language were included (Fig. 1).

The cross-sectional study was conducted in October 2021 using an online platform (Survey Monkey). The survey was distributed to over 230 members of the WHO Health EDRM RN and other experts recommended by the WHO collaboration group on health workforce development [24]. The eligibility criteria included respondents aged 18 or above working in Health EDRM professional development, education, and training programs. The questionnaire (Supplementary file 2) comprised of 28 questions, including agency information, the identification of relevant management and technical competencies, curricula, and evidence gaps. The management and technical competencies included were based on the relevant components in the WHO Health EDRM Framework and reviewed by experts in the investigator team. Ethical approval was obtained from The Chinese University of Hong Kong Survey and Behavioural Research Ethics Committee.

The competency models and curricula identified in the literature review and the cross-sectional survey were compared, and all the competency models and curricula were analyzed. Gap analyses compared the competency models and curricula identified in the literature review and the survey against the Competency and Training Frameworks and principles of Health EDRM.

## Results

### Summary of literature review and cross-sectional survey

#### Literature review

A summary table of the competencies and curricula identified in the literature search is provided in Supplementary file 3. There were 19 articles describing 15 competency models and curricula for health workers and professionals [25–43]. On six occasions, curricula alongside a competency model were listed [25–29, 33–35, 41, 43], on another six occasions, competency models alone were described [32, 36, 38–40, 42]. In the remaining three, only curricula were described [30, 31, 37].

#### Cross-sectional survey

Sixty-five responses were received in the online survey. The response rate was estimated to be 25%, based on the number of invitations sent by email. However, the exact response rate is not known as recipients of the invitation email were encouraged to forward the invitation to other relevant potential respondents. The survey did not generate any additional competency model or curriculum. Table 1 shows the summary profile of the responding agencies. Most respondents were from academic institutions (60%), followed by those from national governments (19%).

**Table 1:** Summary profile of respondents’ agencies (*n*=57)

	n	(%)
**Agency type**		
Academic institution	34	60%
Government	11	19%
Non-governmental organization	4	7%
Inter-governmental organization	3	5%
Private sector	1	2%
Others	4	7%
**Agency level (multiple responses allowed)**		
International	40	70%
National	38	67%
Provincial/ state	21	37%
Local/ municipal	28	49%
Community	25	44%
**Work performed by organization (multiple responses allowed)**		
Research	54	95%
Program development	35	61%
Program implementation	35	61%
Monitoring and evaluation	31	54%
Risk assessment	30	53%
Policy development	28	49%
Others	11	19%
**Agency serving which disaster phase (multiple responses allowed)**		
Prevention	47	82%
Preparedness	54	95%
Readiness	45	79%
Response	45	79%
Recovery	39	68%
**Location where respondent based**		
Japan	5	
UK	4	
US	3	
Australia, Philippines	2	
China, Bahamas, Denmark, France, Ghana, Italy, Malaysia, Nepal, Netherlands, Nigeria, Rwanda, Sri Lanka, Sweden, Switzerland, Thailand	1	

Twenty-one respondents (32%) reported that their agencies had defined the core competencies for Health EDRM managers and frontline responders while 20 respondents (31%) listed the contents in the curricula provided for the managers and frontline responders.

### Competency models from literature review and survey

Among the 12 competency models identified in the literature review, the majority were from the US. Most papers included expert panel reviews while some used the Delphi method for building consensus. Most models were all-hazard in coverage although a few had been developed specifically to counter bioterrorism. Most papers focused on public health workers, but some also targeted public health managers and professionals while others focused on health professionals of various disciplines. Most competency models included knowledge and skills, emergency response systems (including incident management principles), communications, critical thinking, ethical and legal aspects, and managerial and leadership skills. Some competency models provided specific competencies for different tiers of frontline and managerial personnel [25–27, 29, 36].

Table 2 shows the detailed responses from the survey. Management skills were most commonly described, including planning, organizing, applying management processes, establishing effective communication systems, and providing effective leadership (95-100% of respondents). Fewer respondents reported the requirement of management skills for frontline workers, except for effective communication systems (90%).

**Table 2:** Percentage of respondents with management and technical competencies required by their institutions and the coverage from the training curricula provided for Health EDRM managers and frontline personnel

**MANAGEMENT**	Competency models (*n*=21)		Curricula (*n*=20)	
	Managers	Frontline	Managers	Frontline
Planning for health emergency and disaster risk management (across prevention, preparedness, response and recovery)	100%	71%	100%	70%
Organizing health emergency and disaster risk management systems and programs	100%	57%	95%	45%
Effective leadership	100%	67%	100%	65%
Applying management processes	95%	67%	100%	60%
Effective communication systems	100%	90%	100%	85%
**TECHNICAL COMPETENCIES**	Competency model		Curricula	
	Managers	Frontline	Managers	Frontline
Development of Health EDRM policies, strategies and legislation	95%	24%	80%	25%
Health EDRM capacity assessment	100%	71%	90%	60%
Human resource management	100%	57%	100%	55%
Managing coordination mechanisms	95%	67%	100%	70%
Financial resources – planning and managing budgets	95%	24%	85%	30%
Program management	95%	38%	85%	35%
Management of Monitoring and Evaluation systems	95%	43%	90%	45%
Risk assessments	90%	90%	100%	85%
Hazard specific knowledge	86%	95%	95%	90%
Understanding of community vulnerabilities	86%	90%	85%	85%
Managing Health EDRM programs	95%	62%	85%	55%
Preventing emergency and disaster risk	95%	90%	85%	80%
Preparedness and readiness for emergencies and disasters	90%	86%	100%	80%
Managing emergency and disaster response	95%	86%	95%	75%
Managing emergency and disaster recovery	95%	76%	80%	60%
Surge capacity planning	95%	43%	95%	45%
Emergency health/medical teams	86%	81%	85%	75%
Emergency communications	86%	90%	85%	80%
Emergency operations	90%	81%	90%	75%
Logistics and supply systems	90%	67%	85%	65%
Managing information for emergency operations	90%	76%	85%	65%
Managing incident management systems	90%	76%	95%	60%
Managing emergency operations centers	95%	52%	95%	50%
Managing emergency simulations/exercises	95%	67%	95%	70%
Risk communication/communicating with the public	90%	86%	90%	80%
Managing information and communication systems for Health EDRM	90%	71%	80%	55%
Understanding community capacities, leadership and involvement	90%	76%	75%	55%
Cultural competencies	90%	90%	75%	70%
Knowledge of public health principles and practices	90%	81%	90%	80%
Managing health aspects of mass gatherings	90%	86%	95%	75%
Understanding health needs of populations	95%	81%	85%	65%
Understanding healthcare systems and services	90%	76%	90%	65%
Emergency and disaster medical systems	86%	76%	90%	70%
Safe healthcare facilities	90%	62%	95%	50%
Communicable diseases	81%	90%	90%	85%
Disease surveillance	90%	76%	95%	70%
Occupational health and safety	81%	76%	85%	75%
Environmental health	86%	76%	80%	60%
Managing displaced populations	81%	62%	80%	50%

Managers were expected to master a wide range of technical competencies. However, seven items were less frequently included as required competencies for frontline workers (<60% of respondents): 1. human resource management, 2. managing emergency operations centres, 3. managing monitoring and evaluation systems, 4. surge capacity planning, 5. program management, 6. development of Health EDRM policies, strategies and legislation, and 7. financial resources – planning and managing budgets.

### Curricula from the literature review and survey

The majority of the nine identified curricula were from the US. Six articles described a proposed curriculum to address a specific competency model. Structured short training programs like the Core/Basic/Advanced Disaster Life Support and the National Disaster Life Support Decontamination courses [37] were geared towards delivering specific disaster preparedness and response knowledge and skills in 8-16 hours. More extended curricula, including those by CDC/ Columbia University School of Nursing [28, 30, 31, 33], were self-paced and included 15 activities to cover various competencies. Some curricula included exercises and simulation-based training [37, 41, 43].

Three of the six curricula described how the candidates were assessed against defined competencies (Supplementary file 3g). There were no standardized assessment methods: a wide variety of assessments were used, including pre- and post-test scores, self-assessment or trainer-rated performance, exercises (including simulation-based ones) performance results, and observed field-based performance.

Most of the curricula cover the required management and technical competencies across prevention, preparedness, response, and recovery for managers and frontline workers. However, “understanding community capacities, leadership and involvement” and “cultural competencies” were less often covered in curricula (75%). Training delivered by institutions included practical skills training and tabletop/full-scale exercises (85%), followed by blended learning (70%), didactic teaching (65%), online training (65%) and work-based mentorship (55%). Program duration was usually less than one week (40%) or longer than one year (35%). Only 20% were 1-4 weeks and 5% were 1-6 months. Among training modalities that required recertification (55% of the responses), most required recertification every 1-2 years (45%).

### Gaps in competencies and curricula

**Fig. 2:**
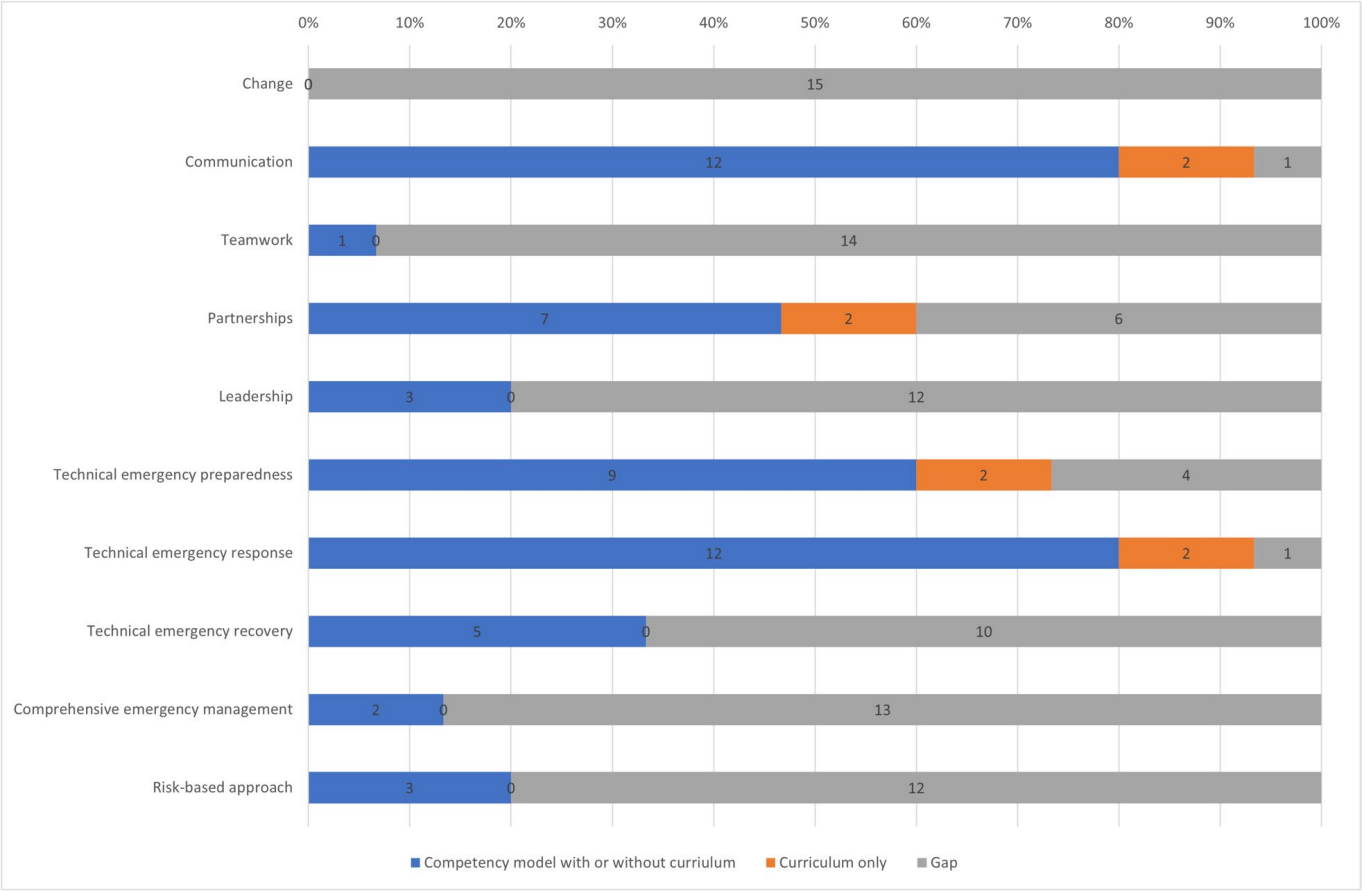
Percentage of published competency models and curricula covering the WHE core competencies and Health EDRM principles

Gaps in competencies and curricula were identified by comparing the above findings with WHE core competencies and Health EDRM principles. WHE core competencies include six areas, namely 1. Moving forward in a changing environment, 2. Applying technical expertise, 3. Communication, 4. Teamwork, 5. Building and promoting partnerships, and 6. Leadership. By comparison, none of the 15 competency models and curricula identified in the literature review and survey included the area of “moving forward in a changing environment,” and only one curriculum covered “teamwork” (Fig. 2, Supplementary file 3d). “Leadership” was only included in three of the 15 competency models and curricula. Most competency models encompassed technical competencies on disaster preparedness and response, but fewer included technical competencies on recovery.

The Health EDRM principle of a comprehensive emergency management perspective (across prevention, preparedness, readiness, response, and recovery) was only included in two models/curricula. The Health EDRM risk-based approach that emphasizes reducing hazards, exposures, and vulnerabilities was only included in three models or curricula (Fig. 2).

The competency models and curricula cited by survey respondents included a higher coverage of the WHE core competencies and the Health EDRM principles than those identified from the literature review (Supplementary 3e and f). However, gaps were still seen in the risk-based approach and technical competencies in emergency recovery and leadership.

### Research priorities for developing Health EDRM competencies (from survey respondents)

Thirty-one survey respondents (48%) provided their views on research priorities for developing Health EDRM competencies in their countries. Effective leadership and planning for Health EDRM were ranked as the most important research priorities for managerial and frontline personnel (Table 3).

**Table 3:** Research priorities in developing Health EDRM competencies in your country for Health EDRM managers and frontline personnel (*n*=31)

**MANAGEMENT**	Managers		Frontline	
	score	%	score	%
Planning for health emergency and disaster risk management (across prevention, preparedness, response and recovery)	112	72%	102	66%
Organizing health emergency and disaster risk management systems and programs	108	70%	93	60%
Effective leadership	112	72%	97	63%
Applying management processes	87	56%	79	51%
Effective communication systems	88	57%	91	59%
**TECHNICAL COMPETENCIES**	Managers		Frontline	
	score	%	score	%
Development of Health EDRM policies, strategies and legislation	101	65%	63	41%
Health EDRM capacity assessment	84	54%	78	50%
Human resource management	89	57%	86	55%
Managing coordination mechanisms	92	59%	82	53%
Financial resources – planning and managing budgets	78	50%	64	41%
Program management	76	49%	79	51%
Management of monitoring and evaluation systems	87	56%	79	51%
Risk assessments	75	48%	74	48%
Hazard specific knowledge	61	39%	85	55%
Understanding of community vulnerabilities	87	56%	85	55%
Managing health EDRM programs	72	46%	64	41%
Preventing emergency and disaster risk	80	52%	73	47%
Preparedness and readiness for emergencies and disasters	79	51%	85	55%
Managing emergency and disaster response	81	52%	87	56%
Managing emergency and disaster recovery	78	50%	73	47%
Surge capacity planning	78	50%	62	40%
Emergency health/medical teams	71	46%	70	45%
Emergency communications	85	55%	86	55%
Emergency operations	80	52%	80	52%
Logistics and supply systems	77	50%	67	43%
Managing information for emergency operations	84	54%	73	47%
Managing incident management systems	74	48%	77	50%
Managing emergency operations centers	76	49%	61	39%
Managing emergency simulations/exercises	68	44%	71	46%
Risk communication/communicating with the public	91	59%	84	54%
Managing information and communication systems for Health EDRM	78	50%	61	39%
Understanding community capacities, leadership and involvement	77	50%	67	43%
Cultural competencies	69	45%	79	51%
Knowledge of public health principles and practices	71	46%	80	52%
Managing health aspects of mass gatherings	67	43%	77	50%
Understanding health needs of populations	74	48%	72	46%
Understanding healthcare systems and services	72	46%	73	47%
Emergency and disaster medical systems	77	50%	84	54%
Safe healthcare facilities	78	50%	73	47%
Communicable diseases	69	45%	81	52%
Disease surveillance	74	48%	75	48%
Occupational health and safety	73	47%	76	49%
Environmental health	65	42%	71	46%
Managing displaced populations	73	47%	63	41%

% calculated out of maximum score possible (155)

## Discussion

The literature review in this study identified studies on competency models and curricula for managers and frontline personnel managing the risks of disasters and emergencies. It was observed that most identified competencies were related to technical aspects of disaster preparedness and response, with fewer focused on recovery. In the WHO Health EDRM Framework, the importance of adopting a comprehensive approach to emergency management was highlighted, covering all aspects from prevention to preparedness, readiness, response, and recovery.

This study found a gap here in most of the current competency models and curricula. This study also discovered no standardized method for assessing competencies while various techniques such as pre- and post-test scores, self-assessment, trainer-rated performance, exercise-based performance results, and field-based performance were used instead.

The cross-sectional survey revealed that Health EDRM agencies increasingly recognize the importance of planning, organizing, and applying risk management systems and programs across prevention, preparedness, response, and recovery. It should be highlighted that different levels of planning (strategic vs operational) might be required for managers and frontline personnel. Similarly, the importance of disaster response planning has been reflected in many competency models and curricula [25–33, 35–37, 39–43]. Besides, Olu et al. also included risk assessments, capacity assessments, and population needs assessments in their competency model, as well as risk reduction implementation and post-emergency health systems recovery [41]. Our survey also confirmed that effective planning across prevention, preparedness, response, and recovery remains an essential priority for research, both for managers and frontline personnel.

Leadership competencies and training constitute another crucial area as reflected in the survey results, as well as being a priority for further research. While the competency of effective organizational management, such as emergency or incident management systems, was well recognized across the literature [25–43], leadership competency goes beyond organizational structures. Olson et al. highlighted leadership and systems thinking in their competency model [35] while Olu et al. included effective leadership, teamwork, and the management skills required for disaster risk management in their model [41].

Decision-making and leadership theories provide the framework supporting the need for leadership competencies [44, 45]. Systems thinking, such as situational awareness, is also a crucial Health EDRM competency for individuals to establish a mental picture of disaster situations [45].

In addition, it is essential to consider the appropriateness of specific competencies for specific organizations. Not all organizations require all of the competencies that have been identified. The WHE Learning Strategy published in 2018 specifically separated functional competencies from core competencies [22]. The WHE core competencies include skills, knowledge, and attributes performance standards across all health emergency workforce, while the functional competencies depend on the specific role of the emergency personnel. The requirement for all WHE personnel to fulfill the behavioral indicators for these core competencies was probably a key driver for this distinction since unlike the universally required Health EDRM core competencies, the functional competencies required will differ between members of the health workforce in different technical roles (depending on the disaster-related or humanitarian work undertaken).

Including attributes, in addition to knowledge and skills, in the WHE core competency framework was a fundamental change. This was highlighted in our study as a lack of coverage of attributes in the models and curricula in the published literature. For example, “moving forward in a changing environment” included indicators such as open-mindedness, a learning mindset, flexibility, and adaptability.

While some of these attributes, such as flexibility and adaptability, have appeared in various current competency models, the emphasis on the inclusion of these new competency standards in staff recruitment, training and assessment, as well as appraisal and performance management in the WHE core competency framework, may help to establish a high-quality workforce for dealing with modern emergencies.

## Limitations

There were several limitations to the literature review and the cross-sectional survey. First of all, since the literature review was conducted before the COVID-19 pandemic, so it cannot reflect the changes during and after the pandemic. Second, although a systematic search strategy was employed to identify articles for this literature review, the methodology of independent screening and systematic data retrieval used in systematic reviews was not adopted in this study. Third, the medical databases selected in this study for literature review may have missed studies in development-related fields and those not published in the academic literature. Therefore, the articles included in this review and the subsequent retrieval of information may be subject to bias. Fourth, many curricula identified in this study did not specify their length of training and thus the effect of training duration on the Health EDRM competencies included in the curricula could not be investigated.

The limitations of the cross-sectional survey included the small sample size and the issue of selection bias due to the way participants were recruited. Concerning the competencies and the curricula cited in the survey, differences could arise due to the specific nature and mandate of the agencies involved in disaster management, whether they be local or international. Furthermore, most respondents did not provide the competencies or curricula in writing and thus the information may be subject to respondent bias. In addition, due to the lack of detail on the competencies and curricula provided, we cannot be sure that the competency models and curricula described in the survey do not overlap with those found in the literature review. Regarding the priorities for research, the respondents’ personal background and experience could have affected the responses.

## Conclusions

Competency mapping in this literature review and cross-sectional survey identified that Health EDRM managers are expected to master many managerial and technical skills while decision-making and leadership skills were increasingly recognized as essential. Future competency models and curricula should separate the general core and the role-specific functional competencies, as well as covering comprehensive core competency domains such as those highlighted by the WHE. A comprehensive risk management framework, such as the WHO Health EDRM Framework, should be adopted in designing both general core and role-specific functional competencies using a Health EDRM risk-based approach.

### Supplementary Information

**Additional file 1: Supplementary file 1.** Search terms in English and Japanese language.

**Additional file 2.** Online questionnaire.

**Additional file 3.** **Supplementary 3a.** Summary table for curricula alongside a competency model identified in English and Japanese language literature review. **Supplementary 3b.** Summary table for competency models identified in English and Japanese language literature review. **Supplementary 3c.** Summary table for curricula (without a competency model) identified in English and Japanese language literature review. **Supplementary 3d.** Gap analysis of published competency model and curricula against the WHO Health Emergency Programme Core Competencies and health EDRM perspectives. **Supplementary 3e.** Gap analysis of survey competency model against selected WHO Health Emergency Programme Core Competencies and health EDRM perspectives. **Supplementary 3f.** Gap analysis of survey curricula against selected WHO Health Emergency Programme Core Competencies and health EDRM perspectives. **Supplementary 3g.** Assessment of competency attainment in published curricula with a competency model. **Supplementary 3h.** Knowledge and skills listed by survey respondents not included in the list of management and technical competencies.

## Data Availability

The datasets generated and/or analysed during the current study are available from the corresponding author on reasonable request.

## Abbreviations

CASK model: Competency = Attributes + Skills + Knowledge model
CBRN: Chemical, biological, radiological and nuclear
CDC: Centers for Disease Control and Prevention, United States
Health EDRM: Health emergency and disaster risk management
Health EDRM RN: WHO Health EDRM Research Network
US: United States
WHE: WHO Health Emergencies Programme
WHO: World Health Organization
