# Correction: Mapping study for health emergency and disaster risk management competencies and curricula: literature review and cross-sectional survey

**DOI:** 10.1186/s12992-024-01037-9

**Published:** 2024-04-12

**Authors:** Kevin K. C. Hung, Makiko K. MacDermot, Theresa S. I. Hui, Suet Yi Chan, Sonoe Mashino, Catherine P. Y. Mok, Pak Ho Leung, Ryoma Kayano, Jonathan Abrahams, Chi Shing Wong, Emily Y. Y. Chan, Colin A. Graham

**Affiliations:** 1 Accident and Emergency Medicine Academic Unit, Trauma & Emergency Centre, The Chinese University of Hong Kong, Prince of Wales Hospital, New Territories, Shatin, Hong Kong; 2 Collaborating Centre for Oxford University, JC School of Public Health and Primary Care, Chinese University of Hong Kong for Disaster and Medical Humanitarian Response (CCOUC), The Chinese University of Hong Kong, Shatin, Hong Kong China; 3 Research Institute of Nursing Care for People and Community, University of Hyogo, 673-8588 Akashi, Japan; 4 World Health Organization, Centre for Health Development, 651-0073 Kobe, Japan; 5 Disaster Resilience Initiative, Monash University, Monash University, Clayton, Australia

**Globalization and Health (2024) 20:15**

10.1186/s12992-023-01010-y

Following publication of the original article, it was brought to the journal's attention that the article had published with the wrong license: it had published with the Creative Commons Attribution 4.0 International License, whereas the correct license is the Creative Commons Attribution 3.0 IGO License. The license has been corrected in the published article. The publisher thanks you for reading this erratum and apologizes for any inconvenience caused.

