# Supplementary material for: Mapping study for health emergency and disaster risk management competencies and curricula: literature review and cross-sectional survey
Source: Global Health. 2024 Feb 21;20:15. doi: 10.1186/s12992-023-01010-y (PMC10880341; doi:10.1186/s12992-023-01010-y)
Supplement: Supplementary file 1 — Additional file 1: Supplementary file 1. Search terms in English and Japanese language. [file 12992_2023_1010_MOESM1_ESM.docx]

Supplementary file 1: Search terms in English and Japanese language

For the English language literature review, English language literature published from 1990 to 11 March 2020 was conducted using MEDLINE (1966), EMBASE (1980) and CINAHL (1980). The English language search terms were as follows:

(*Disaster*.tw* ***OR*** *Public health emergenc*.tw* ***OR*** *Cris?s.tw* ***OR*** *Humanitarian.tw* ***OR*** *Complex emergenc*.tw* ***OR*** *Outbreak*.tw* ***OR*** *War.tw* ***OR*** *Conflict.tw)*

***AND***

*(Exp Workforce* ***OR*** *Exp Health Personnel* ***OR*** *Exp Emergency Responders* ***OR*** *Exp Volunteers* ***OR*** *Exp Personnel Management* ***OR*** *Exp Surge Capacity* ***OR*** *Exp Education* ***OR*** *Exp Quality of Health Care* ***OR*** *Exp Credentialing*)

For the Japanese language literature review, we searched for articles written in Japanese by using the ICHUSHI database for the period from 1990 to 23 October 2020. The search terms below included (“disaster” or “health emergency”) and (“education” or “training”) related Japanese words were used as following:

(((((養成/AL) and (AB=Y and PT=会議録除く)) and ((((災害/TH or 災害/AL)) and (AB=Y and PT=会議録除く)) or ((健康危機/AL) and (AB=Y and PT=会議録除く)))) or ((((教育/TH or 教育/AL)) and (AB=Y and PT=会議録除く)) and ((((災害/TH or 災害/AL)) and (AB=Y and PT=会議録除く)) or ((健康危機/AL) and (AB=Y and PT=会議録除く)))) or (((育成/AL) and (AB=Y and PT=会議録除く)) and ((((災害/TH or 災害/AL)) and (AB=Y and PT=会議録除く)) or ((健康危機/AL) and (AB=Y and PT=会議録除く)))) or ((((体育とトレーニング/TH or 訓練/AL)) and (AB=Y and PT=会議録除く)) and ((((災害/TH or 災害/AL)) and (AB=Y and PT=会議録除く)) or ((健康危機/AL) and (AB=Y and PT=会議録除く)))))) not (((整形外科/TH or 整形外科/AL)) and (AB=Y and PT=会議録除く)) .
