# Supplementary material for: Mapping study for health emergency and disaster risk management competencies and curricula: literature review and cross-sectional survey
Source: Global Health. 2024 Feb 21;20:15. doi: 10.1186/s12992-023-01010-y (PMC10880341; doi:10.1186/s12992-023-01010-y)
Supplement: Supplementary file 2 — Additional file 2. Online questionnaire. [file 12992_2023_1010_MOESM2_ESM.docx]

**Online questionnaire**

**Evidence Mapping for Professional Development Programmes and Courses in Health Emergency Disaster Risk Management (Health EDRM)**

**Information page and consent**

**Background**

This survey aims to identify the types of competencies and training that people regard as essential in a comprehensive professional development programme with a focus on Health EDRM managers/ frontline personnel. The survey will also identify gaps in knowledge and future research needs.

This study is part of the World Health Organization (WHO) Centre for Health Development Health EDRM Research Area 4. Ethical approval has been obtained from CUHK Survey and Behavioral Ethics Committee (PI: Dr Kevin Hung). Please contact ccouc@cuhk.edu.hk if you have any questions. Further information can be found at:

https://extranet.who.int/kobe_centre/en/project-details/h-edrm_area4

**Participating in this survey**

This contains 15-20 questions with a combination of open-ended questions and multiple-choice questions which should take no more than 20 minutes to complete.

The planned survey is administered at a global level. Before you begin, we would like to confirm that you are

- ≥ 18 years of age
- have relevant experience in health emergency and disaster risk reduction and in disaster education and training programmes

Please complete the survey within 4 weeks. Your responses will be treated anonymously and be used only for the purpose of our study. The data will be analyzed and disseminated in a report to the WHO to contribute to their knowledge synthesis for Health EDRM, and may be published in a peer reviewed paper in due course.
 
Thank you for your participation in advance!

Q1. Do you consent to participating in this survey?

- Yes
- No

1. **Your Current Work (5 questions)**

Q2. What is the name of your organization you work for?

Response: _____________________________________

Q3. Which category does your organization fall under? (Choose the one that best describes)

- Civil society
- Government
- Academic institution
- Non-governmental Organization
- Private sector
- Inter-governmental Organization
- Other: ____________________________________

Q4. Which level does your organization serve? Select all that apply.

- Community
- Local/Municipal
- Provincial/State level
- National
- International

Q5. Does your organization have a role in the following? Select all that apply.

- Prevention
- Preparedness
- Readiness
- Response
- Recovery

Q6. How would you describe the work that your organization performs? Select all the apply

- Programme development
- Programme implementation
- Policy development
- Risk assessment
- Research
- Monitor and evaluation
- Other (please specify): ____________________________________________

**Part 1 Continue**

*“Health EDRM is the systematic analysis and management of health risks, posed by actual or potential hazardous events, including emergencies and disasters, through a combination of hazard, exposure and vulnerability reduction to prevent and mitigate risks, preparedness, response, and recovery.”*

Extracted from GLOSSARY of Health Emergency and Disaster Risk Management Terminology.

Q7. Are there any managers/frontline personnel responsible for Health EDRM in your organization?

- Yes
- No

1. **Health EDRM manager/frontline personnel competencies in your organization (4 questions)**

In our context, a competency is defined as:

"the ability to apply knowledge and skills to achieve intended results.”

ISO 22300:2018 Security and resilience—Vocabulary. International Organization for Standardization, Geneva (https://www.iso.org/standard/68436.html)

Q8. My organization uses competency-based assessment in Health EDRM for our managers or frontline personnel.

- Yes
- No

**Part 2 continue**

Q9. If it is possible to share, please attach your competency matrix or other documents outlining how competency is determined in your organization from your organization. (We will anonymize this information.)

Choose File: ______________________________________________

Q10. How did your organization design this competency matrix?

- Designed internally without any external references
- Used evidence (research/recommendations) from external sources.

Please specify: ________________

- I am not sure.

Q11. What are the basic knowledge and skills to be competent in managing the risks associated with emergencies and disasters? (Select all that apply.)

|  | **Managers** | **Frontline personnel** |
| --- | --- | --- |
| **MANAGEMENT** |  |  |
| Planning for health emergency and disaster risk management (across prevention, preparedness, response and recovery) |  |  |
| Organizing health emergency and disaster risk management systems and programs |  |  |
| Effective leadership |  |  |
| Applying management processes |  |  |
| Effective communication systems |  |  |
|  |  |  |
| **TECHNICAL COMPETENCIES** |  |  |
| Development of Health EDRM Policies, Strategies and Legislation |  |  |
| Health EDRM capacity assessment |  |  |
| Human Resource Management |  |  |
| Managing Coordination Mechanisms |  |  |
| Financial resources – planning and managing budgets |  |  |
| Programme management |  |  |
| Management of Monitoring and Evaluation systems |  |  |
| Risk assessments |  |  |
| Hazard specific knowledge |  |  |
| Understanding of community vulnerabilities |  |  |
| Managing health EDRM programmes |  |  |
| Preventing emergency and disaster risk |  |  |
| Preparedness and readiness for emergencies and disasters |  |  |
| Managing emergency and disaster response |  |  |
| Managing emergency and disaster recovery |  |  |
| Surge capacity planning |  |  |
| Emergency health/medical teams |  |  |
| Emergency Communications |  |  |
| Emergency Operations |  |  |
| Logistics and supply systems |  |  |
| Managing information for Emergency Operations |  |  |
| Managing Incident Management Systems |  |  |
| Managing Emergency Operations Centers |  |  |
| Managing Emergency Simulations/Exercises |  |  |
| Risk communication/Communicating with the Public |  |  |
| Managing Information and Communication Systems for Health EDRM |  |  |
| Understanding Community Capacities, Leadership and Involvement |  |  |
| Cultural competencies |  |  |
| Knowledge of public health principles and practices |  |  |
| Managing Health Aspects of Mass Gatherings |  |  |
| Understanding Health Needs of Populations |  |  |
| Understanding Healthcare Systems and Services |  |  |
| Emergency and Disaster Medical Systems |  |  |
| Safe Healthcare Facilities |  |  |
| Communicable Diseases |  |  |
| Disease Surveillance |  |  |
| Occupational Health and Safety |  |  |
| Environmental Health |  |  |
| Managing Displaced Populations |  |  |
| Others (please specify): ___________________________ |  |  |

1. **Health EDRM manager training in your organization (6 questions)**

Q12. Does your organization provide access to in-house or external professional development for managers responsible for/involved in Health EDRM?

- Yes
- No

**Part 3 continue**

Q13. If it is possible to share, please attach any curriculums/programmes (if available) or any outline of any curriculums/programmes provided from your organization. (We will anonymize this information.)

Choose File: ___________________________

Q14. Do in-house curriculums/programmes or any required curriculums/programmes in your organization contain any of the following? (Select all that apply.)

|  | **Managers** | **Frontline personnel** |
| --- | --- | --- |
| Same as the answer provided in previous response (in part ii competencies) |  |  |
| **If no, please select from below:** |  |  |
|  |  |  |
| **MANAGEMENT** |  |  |
| Planning for health emergency and disaster risk management (across prevention, preparedness, response and recovery) |  |  |
| Organizing health emergency and disaster risk management systems and programs |  |  |
| Effective leadership |  |  |
| Applying management processes |  |  |
| Effective communication systems |  |  |
|  |  |  |
| **TECHNICAL COMPETENCIES** |  |  |
| Development of Health EDRM Policies, Strategies and Legislation |  |  |
| Health EDRM capacity assessment |  |  |
| Human Resource Management |  |  |
| Managing Coordination Mechanisms |  |  |
| Financial resources – planning and managing budgets |  |  |
| Programme management |  |  |
| Management of Monitoring and Evaluation systems |  |  |
| Risk assessments |  |  |
| Hazard specific knowledge |  |  |
| Understanding of community vulnerabilities |  |  |
| Managing health EDRM programmes |  |  |
| Preventing emergency and disaster risk |  |  |
| Preparedness and readiness for emergencies and disasters |  |  |
| Managing emergency and disaster response |  |  |
| Managing emergency and disaster recovery |  |  |
| Surge capacity planning |  |  |
| Emergency health/medical teams |  |  |
| Emergency Communications |  |  |
| Emergency Operations |  |  |
| Logistics and supply systems |  |  |
| Managing information for Emergency Operations |  |  |
| Managing Incident Management Systems |  |  |
| Managing Emergency Operations Centers |  |  |
| Managing Emergency Simulations/Exercises |  |  |
| Risk communication/Communicating with the Public |  |  |
| Managing Information and Communication Systems for Health EDRM |  |  |
| Understanding Community Capacities, Leadership and Involvement |  |  |
| Cultural competencies |  |  |
| Knowledge of public health principles and practices |  |  |
| Managing Health Aspects of Mass Gatherings |  |  |
| Understanding Health Needs of Populations |  |  |
| Understanding Healthcare Systems and Services |  |  |
| Emergency and Disaster Medical Systems |  |  |
| Safe Healthcare Facilities |  |  |
| Communicable Diseases |  |  |
| Disease Surveillance |  |  |
| Occupational Health and Safety |  |  |
| Environmental Health |  |  |
| Managing Displaced Populations |  |  |
| Others (please specify): ___________________________ |  |  |

Q15. How did your organization design the curriculum?

- Designed internally without any external references
- Used evidence (research/recommendations) from external sources.

Please specify: __________________________________________

- I am not sure.

Q16. How are these professional development programmes delivered to colleagues in your organization? (Select all that apply.)

- Didactic
- Online
- Practical skills training
- Table top/full scale exercise
- Blended learning
- Work based mentorship
- Other (please specify): ______________________________________________

Q17. What is the length of the programme?

- Less than 1 week
- 1-4 weeks
- 1-6 months
- 7-12 months
- More than 1 year

Q18. Is recertification required regularly?

- Yes
- No

Q19. If so, how frequent?

- Every 6 months or less
- 7-12 months
- 13 months – 2 years
- More than 2 years

Q20. How did your organization decide on the delivery methods?

- Designed internally without any external references
- Used evidence (research/recommendations) from external sources.

Please specify: __________________________________________

- I am not sure.

1. **Your view towards the future research for health EDRM education (2 questions)**

Q21. In your view, where do you think the priorities for future research lie in developing Health EDRM competencies in your country? Please rank 1^st^ to 5^th^ priority. (Please select at least one option each for managers AND frontline personnel.)

|  | **Managers** | **Frontline personnel** |
| --- | --- | --- |
| **MANAGEMENT** |  |  |
| Planning for health emergency and disaster risk management (across prevention, preparedness, response and recovery) | 1^st^ priority  2^nd^ priority  3^rd^ priority  4^th^ priority  5^th^ priority | 1^st^ priority  2^nd^ priority  3^rd^ priority  4^th^ priority  5^th^ priority |
| Organizing health emergency and disaster risk management systems and programs | 1^st^ priority  2^nd^ priority  3^rd^ priority  4^th^ priority  5^th^ priority | 1^st^ priority  2^nd^ priority  3^rd^ priority  4^th^ priority  5^th^ priority |
| Effective leadership | 1^st^ priority  2^nd^ priority  3^rd^ priority  4^th^ priority  5^th^ priority | 1^st^ priority  2^nd^ priority  3^rd^ priority  4^th^ priority  5^th^ priority |
| Applying management processes | 1^st^ priority  2^nd^ priority  3^rd^ priority  4^th^ priority  5^th^ priority | 1^st^ priority  2^nd^ priority  3^rd^ priority  4^th^ priority  5^th^ priority |
| Effective communication systems | 1^st^ priority  2^nd^ priority  3^rd^ priority  4^th^ priority  5^th^ priority | 1^st^ priority  2^nd^ priority  3^rd^ priority  4^th^ priority  5^th^ priority |
|  |  |  |
| **TECHNICAL COMPETENCIES** |  |  |
| Development of Health EDRM Policies, Strategies and Legislation | 1^st^ priority  2^nd^ priority  3^rd^ priority  4^th^ priority  5^th^ priority | 1^st^ priority  2^nd^ priority  3^rd^ priority  4^th^ priority  5^th^ priority |
| Health EDRM capacity assessment | 1^st^ priority  2^nd^ priority  3^rd^ priority  4^th^ priority  5^th^ priority | 1^st^ priority  2^nd^ priority  3^rd^ priority  4^th^ priority  5^th^ priority |
| Human Resource Management | 1^st^ priority  2^nd^ priority  3^rd^ priority  4^th^ priority  5^th^ priority | 1^st^ priority  2^nd^ priority  3^rd^ priority  4^th^ priority  5^th^ priority |
| Managing Coordination Mechanisms | 1^st^ priority  2^nd^ priority  3^rd^ priority  4^th^ priority  5^th^ priority | 1^st^ priority  2^nd^ priority  3^rd^ priority  4^th^ priority  5^th^ priority |
| Financial resources – planning and managing budgets | 1^st^ priority  2^nd^ priority  3^rd^ priority  4^th^ priority  5^th^ priority | 1^st^ priority  2^nd^ priority  3^rd^ priority  4^th^ priority  5^th^ priority |
| Programme management | 1^st^ priority  2^nd^ priority  3^rd^ priority  4^th^ priority  5^th^ priority | 1^st^ priority  2^nd^ priority  3^rd^ priority  4^th^ priority  5^th^ priority |
| Management of Monitoring and Evaluation systems | 1^st^ priority  2^nd^ priority  3^rd^ priority  4^th^ priority  5^th^ priority | 1^st^ priority  2^nd^ priority  3^rd^ priority  4^th^ priority  5^th^ priority |
| Risk assessments | 1^st^ priority  2^nd^ priority  3^rd^ priority  4^th^ priority  5^th^ priority | 1^st^ priority  2^nd^ priority  3^rd^ priority  4^th^ priority  5^th^ priority |
| Hazard specific knowledge | 1^st^ priority  2^nd^ priority  3^rd^ priority  4^th^ priority  5^th^ priority | 1^st^ priority  2^nd^ priority  3^rd^ priority  4^th^ priority  5^th^ priority |
| Understanding of community vulnerabilities | 1^st^ priority  2^nd^ priority  3^rd^ priority  4^th^ priority  5^th^ priority | 1^st^ priority  2^nd^ priority  3^rd^ priority  4^th^ priority  5^th^ priority |
| Managing health EDRM programmes | 1^st^ priority  2^nd^ priority  3^rd^ priority  4^th^ priority  5^th^ priority | 1^st^ priority  2^nd^ priority  3^rd^ priority  4^th^ priority  5^th^ priority |
| Preventing emergency and disaster risk | 1^st^ priority  2^nd^ priority  3^rd^ priority  4^th^ priority  5^th^ priority | 1^st^ priority  2^nd^ priority  3^rd^ priority  4^th^ priority  5^th^ priority |
| Preparedness and readiness for emergencies and disasters | 1^st^ priority  2^nd^ priority  3^rd^ priority  4^th^ priority  5^th^ priority | 1^st^ priority  2^nd^ priority  3^rd^ priority  4^th^ priority  5^th^ priority |
| Managing emergency and disaster response | 1^st^ priority  2^nd^ priority  3^rd^ priority  4^th^ priority  5^th^ priority | 1^st^ priority  2^nd^ priority  3^rd^ priority  4^th^ priority  5^th^ priority |
| Managing emergency and disaster recovery | 1^st^ priority  2^nd^ priority  3^rd^ priority  4^th^ priority  5^th^ priority | 1^st^ priority  2^nd^ priority  3^rd^ priority  4^th^ priority  5^th^ priority |
| Surge capacity planning | 1^st^ priority  2^nd^ priority  3^rd^ priority  4^th^ priority  5^th^ priority | 1^st^ priority  2^nd^ priority  3^rd^ priority  4^th^ priority  5^th^ priority |
| Emergency health/medical teams | 1^st^ priority  2^nd^ priority  3^rd^ priority  4^th^ priority  5^th^ priority | 1^st^ priority  2^nd^ priority  3^rd^ priority  4^th^ priority  5^th^ priority |
| Emergency Communications | 1^st^ priority  2^nd^ priority  3^rd^ priority  4^th^ priority  5^th^ priority | 1^st^ priority  2^nd^ priority  3^rd^ priority  4^th^ priority  5^th^ priority |
| Emergency Operations | 1^st^ priority  2^nd^ priority  3^rd^ priority  4^th^ priority  5^th^ priority | 1^st^ priority  2^nd^ priority  3^rd^ priority  4^th^ priority  5^th^ priority |
| Logistics and supply systems | 1^st^ priority  2^nd^ priority  3^rd^ priority  4^th^ priority  5^th^ priority | 1^st^ priority  2^nd^ priority  3^rd^ priority  4^th^ priority  5^th^ priority |
| Managing information for Emergency Operations | 1^st^ priority  2^nd^ priority  3^rd^ priority  4^th^ priority  5^th^ priority | 1^st^ priority  2^nd^ priority  3^rd^ priority  4^th^ priority  5^th^ priority |
| Managing Incident Management Systems | 1^st^ priority  2^nd^ priority  3^rd^ priority  4^th^ priority  5^th^ priority | 1^st^ priority  2^nd^ priority  3^rd^ priority  4^th^ priority  5^th^ priority |
| Managing Emergency Operations Centres | 1^st^ priority  2^nd^ priority  3^rd^ priority  4^th^ priority  5^th^ priority | 1^st^ priority  2^nd^ priority  3^rd^ priority  4^th^ priority  5^th^ priority |
| Managing Emergency Simulations/Exercises | 1^st^ priority  2^nd^ priority  3^rd^ priority  4^th^ priority  5^th^ priority | 1^st^ priority  2^nd^ priority  3^rd^ priority  4^th^ priority  5^th^ priority |
| Risk communication/Communicating with the Public | 1^st^ priority  2^nd^ priority  3^rd^ priority  4^th^ priority  5^th^ priority | 1^st^ priority  2^nd^ priority  3^rd^ priority  4^th^ priority  5^th^ priority |
| Managing Information and Communication Systems for Health EDRM | 1^st^ priority  2^nd^ priority  3^rd^ priority  4^th^ priority  5^th^ priority | 1^st^ priority  2^nd^ priority  3^rd^ priority  4^th^ priority  5^th^ priority |
| Understanding Community Capacities, Leadership and Involvement | 1^st^ priority  2^nd^ priority  3^rd^ priority  4^th^ priority  5^th^ priority | 1^st^ priority  2^nd^ priority  3^rd^ priority  4^th^ priority  5^th^ priority |
| Cultural competencies | 1^st^ priority  2^nd^ priority  3^rd^ priority  4^th^ priority  5^th^ priority | 1^st^ priority  2^nd^ priority  3^rd^ priority  4^th^ priority  5^th^ priority |
| Knowledge of public health principles and practices | 1^st^ priority  2^nd^ priority  3^rd^ priority  4^th^ priority  5^th^ priority | 1^st^ priority  2^nd^ priority  3^rd^ priority  4^th^ priority  5^th^ priority |
| Managing Health Aspects of Mass Gatherings | 1^st^ priority  2^nd^ priority  3^rd^ priority  4^th^ priority  5^th^ priority | 1^st^ priority  2^nd^ priority  3^rd^ priority  4^th^ priority  5^th^ priority |
| Understanding Health Needs of Populations | 1^st^ priority  2^nd^ priority  3^rd^ priority  4^th^ priority  5^th^ priority | 1^st^ priority  2^nd^ priority  3^rd^ priority  4^th^ priority  5^th^ priority |
| Understanding Healthcare Systems and Services | 1^st^ priority  2^nd^ priority  3^rd^ priority  4^th^ priority  5^th^ priority | 1^st^ priority  2^nd^ priority  3^rd^ priority  4^th^ priority  5^th^ priority |
| Emergency and Disaster Medical Systems | 1^st^ priority  2^nd^ priority  3^rd^ priority  4^th^ priority  5^th^ priority | 1^st^ priority  2^nd^ priority  3^rd^ priority  4^th^ priority  5^th^ priority |
| Safe Healthcare Facilities | 1^st^ priority  2^nd^ priority  3^rd^ priority  4^th^ priority  5^th^ priority | 1^st^ priority  2^nd^ priority  3^rd^ priority  4^th^ priority  5^th^ priority |
| Communicable Diseases | 1^st^ priority  2^nd^ priority  3^rd^ priority  4^th^ priority  5^th^ priority | 1^st^ priority  2^nd^ priority  3^rd^ priority  4^th^ priority  5^th^ priority |
| Disease Surveillance | 1^st^ priority  2^nd^ priority  3^rd^ priority  4^th^ priority  5^th^ priority | 1^st^ priority  2^nd^ priority  3^rd^ priority  4^th^ priority  5^th^ priority |
| Occupational Health and Safety | 1^st^ priority  2^nd^ priority  3^rd^ priority  4^th^ priority  5^th^ priority | 1^st^ priority  2^nd^ priority  3^rd^ priority  4^th^ priority  5^th^ priority |
| Environmental Health | 1^st^ priority  2^nd^ priority  3^rd^ priority  4^th^ priority  5^th^ priority | 1^st^ priority  2^nd^ priority  3^rd^ priority  4^th^ priority  5^th^ priority |
| Managing Displaced Populations | 1^st^ priority  2^nd^ priority  3^rd^ priority  4^th^ priority  5^th^ priority | 1^st^ priority  2^nd^ priority  3^rd^ priority  4^th^ priority  5^th^ priority |
| Others (please specify): ___________________________ | 1^st^ priority  2^nd^ priority  3^rd^ priority  4^th^ priority  5^th^ priority | 1^st^ priority  2^nd^ priority  3^rd^ priority  4^th^ priority  5^th^ priority |

Q22. What specific research topic you would like to see that could help improve health EDRM education in the future?

Please specify: ________________________________________________________________

**Participant information (5 questions)**

Q23. What is your age?

- 18-24
- 25-34
- 35-44
- 45-54
- 55-64
- 65+

Q24. What is your gender?

- Female
- Male
- Wish not to identify as male or female

Q25. In what country do you work?

| - Afghanistan - Albania - Algeria - Andorra - Angola - Antigua and Barbuda - Argentina - Armenia - Australia - Austria - Azerbaijan - Bahamas - Bahrain - Bangladesh - Barbados - Belarus - Belgium - Belize - Benin - Bhutan - Bolivia (Plurinational State of) - Bosnia and Herzegovina - Botswana - Brazil - Brunei Darussalam - Bulgaria - Burkina Faso - Burundi - Cabo Verde - Cambodia - Cameroon - Canada - Central African Republic - Chad - Chile - China - Colombia - Comoros - Congo - Costa Rica - Côte D'Ivoire - Croatia - Cuba - Cyprus - Czech Republic - Democratic People's Republic of Korea - Democratic Republic of the Congo - Denmark - Djibouti - Dominica - Dominican Republic - Ecuador - Egypt - El Salvador - Equatorial Guinea - Eritrea - Estonia - Ethiopia - Fiji - Finland - France - Gabon - Gambia - Georgia - Germany - Ghana - Greece - Grenada - Guatemala - Guinea - Guinea Bissau - Guyana - Haiti - Holy See - Honduras - Hungary - Iceland - India - Indonesia - Iran (Islamic Republic of) - Iraq - Ireland - Israel - Italy - Jamaica - Japan - Jordan - Kazakhstan - Kenya - Kiribati - Kuwait - Kyrgyzstan - Lao People’s Democratic Republic - Latvia - Lebanon - Lesotho - Liberia - Libya - Liechtenstein - Lithuania - Luxembourg - Madagascar - Malawi - Malaysia - Maldives - Mali - Malta - Marshall Islands - Mauritania - Mauritius - Mexico - Micronesia (Federated States of) - Monaco - Mongolia - Montenegro - Morocco - Mozambique - Myanmar - Namibia - Nauru - Nepal - Netherlands - New Zealand - Nicaragua - Niger - Nigeria - Norway - Oman - Pakistan - Palau - Panama - Papua New Guinea - Paraguay - Peru - Philippines - Poland - Portugal - Qatar - Republic of Korea - Republic of Moldova - Romania - Russian Federation - Rwanda - Saint Kitts and Nevis - Saint Lucia - Saint Vincent and the Grenadines - Samoa - San Marino - Sao Tome and Principe - Saudi Arabia - Senegal - Serbia - Seychelles - Sierra Leone - Singapore - Slovakia - Slovenia - Solomon Islands - Somalia - South Africa - South ‎Sudan - Spain - Sri Lanka - State of Palestine - Sudan - Suriname - Swaziland - Sweden - Switzerland - Syrian Arab Republic - Tajikistan - Thailand - The former Yugoslav Republic of Macedonia - Timor-Leste - Togo - Tonga - Trinidad and Tobago - Tunisia - Turkey - Turkmenistan - Tuvalu - Uganda - Ukraine - United Arab Emirates - United Kingdom of Great Britain and Northern Ireland - United Republic of Tanzania - United States of America - Uruguay - Uzbekistan - Vanuatu - Venezuela (Bolivarian Republic of) - Vietnam - Yemen - Zambia - Zimbabwe |
| --- |
|  |

Q26. What is your profession? Select all that apply.

- Academic (University based)
- Research
- Policy maker (government)
- Manager (government)
- Technical officer (government)
- Professional/practitioner
- Other (please specify): _____________________________________________________

Q27. About how many years have you served in your organization?

- Less than 1 year
- At least 1 year but less than 3 years
- At least 3 years but less than 5 years
- At least 5 years but less than 10 years
- 10 years or more

**You have completed the survey. Thank you!**
