# Supplementary material for: Mapping study for health emergency and disaster risk management competencies and curricula: literature review and cross-sectional survey
Source: Global Health. 2024 Feb 21;20:15. doi: 10.1186/s12992-023-01010-y (PMC10880341; doi:10.1186/s12992-023-01010-y)
Supplement: Supplementary file 3 — Additional file 3. Supplementary 3a. Summary table for curricula alongside a competency model identified in English and Japanese language literature review. Supplementary 3b. Summary table for competency models identified in English and Japanese language literature review. Supplementary 3c. Summary table for curricula (without a competency model) identified in English and Japanese language literature review. Supplementary 3d. Gap analysis of published competency model and curricula against the WHO Health Emergency Programme Core Competencies and health EDRM perspectives. Supplementary 3e. Gap analysis of survey competency model against selected WHO Health Emergency Programme Core Competencies and health EDRM perspectives. Supplementary 3f. Gap analysis of survey curricula against selected WHO Health Emergency Programme Core Competencies and health EDRM perspectives. Supplementary 3g. Assessment of competency attainment in published curricula with a competency model. Supplementary 3h. Knowledge and skills listed by survey respondents not included in the list of management and technical competencies. [file 12992_2023_1010_MOESM3_ESM.docx]

Supplementary 3a: Summary table for curricula alongside a competency model identified in English and Japanese language literature review

| Author, Year, Country | Type of article | Hazard | Target sample | Content |
| --- | --- | --- | --- | --- |
| Gebbie et al, 2002, USA [25]  Gebbie et al, 2002, USA [26]  Centers for Disease Control and Prevention, 2002, USA [27]  Barnett et al, 2005, USA [28]  Parker et al, 2005, USA [29] | Delphi method with competency assessment  A pilot curriculum with 15 activities based on core competency developed  A pilot training programme using gaming | All-hazard and bioterrorism | Public health frontline and managers | **Competency model and curriculum**  **Road to Preparedness activities corresponding to core competency by Columbia University or organizational readiness challenges**  Competency 1: Describe public health role in emergency response in a range of emergencies that might arise.  • Activity: Learn about public health role in emergency response. Take Columbia online course or equivalent.  Competency 2: Describe chain of command in emergency response.  • Activity: Using templates provided by your agency, write your name in proper place in your agency’s incident command system chain of command.  Competency 3: Identify and locate agency emergency response plan.  • Activity: Identify and locate health department’s emergency response plan or section relevant to your role.  Competency 4: Describe his/her functional role(s) in emergency response and demonstrate his/her role(s) in regular drills.  • Activity: Participate in a training session about what your specific primary and alternate roles would be during an emergency, and why it is important to success of the effort. Identify limits to your own authority.  Competency 5: Demonstrate correct use of all communication equipment used for emergency communication.  • Activity: Demonstrate use of communication equipment.  Competency 6: Describe communication role(s) in emergency response: Within the agency using established communication systems/ With the media/ With the general public / Personal (with family, neighbours)  • Activity: Attend an agency-sponsored lecture, “How to Talk to People about Disasters’’  • Activity: Participate in communication practice session and practice the skills learned during the risk communication lecture, or make a presentation to others about how to talk to people about disasters.  • Activity: Describe your risk communication roles within the agency, with the media, with the general public, and with family/personal contacts.  Competency 7: Identify limits to own knowledge/skill/ authority, and identify key system resources for referring matters that exceed these limits.  • Activity: Participate in a training session about what your specific primary and alternate roles would be during an emergency, and why it is important to the success of the effort. Identify limits to your own authority.  Competency 8: Recognize unusual events that might indicate an emergency and describe appropriate action.  • Activity: Participate in agency-led discussion to explore what kinds of unusual events or trends might indicate an emergency or disaster, and describe appropriate actions.  • Activity: Participate in a training session about weapons of mass destruction and appropriate public health response activities  Competency 9: Apply creative problem solving and flexible thinking to unusual challenges within his/her functional responsibilities and evaluate effectiveness of all actions taken.  • Activity: Participate in exercise/training in which you demonstrate creative problem solving to unusual challenges.  Organizational readiness challenges 1: Promoting worksite safety and security in emergencies  • Activity: Describe how you are participating in the planning and implementation of a plan at your worksite, to ensure the safety and security of staff and clients in the event of an emergency.  Organizational readiness challenges 2: Enhancing personal and family emergency preparedness  • Activity: Write out your own personal and family emergency response plan. Include enough detail that someone else looking at your plan would be able to follow it accurately. Share the plan with your family and everyone involved in carrying out your plan.  Organizational readiness challenges 3: Facilitating capacity to report to duty in a crisis  • Activity: Draw a map showing how you would get from your home to your designated emergency assignment location(s). Plan three different routes in case unexpected street closures obstruct your way.  • Activity: Indicate ways in which you will be notified in the event of an emergency.  **A curriculum with 15 activities (Road Map to Preparedness) based on Core competency developed by Columbia University School of Nursing and Health Policy**    1. Learn about the public health role in emergency response. Take the Columbia online course or equivalent. http://cds.osr.columbia.edu/bepcourse/test.asp  2. Participate in a training about Weapons of Mass Destruction–Online or Agency Sponsored http://www.jhsph.edu/CPHP/Training/Online%20Training/intro_to_wmd.html  3. Participate in a training about what your specific primary and alternate roles would be during an emergency and why it is important to the success of the effort. Identify the limits to your own authority.  4. Describe how you are participating in the planning and implementation of a plan at your worksite to ensure the safety and security of staff and clients in the event of an emergency.  5. Incident Command Using the templates provided, write your name in the proper place in the chain of command.  6. Identify and locate the Health Department’s emergency response plan or the section relevant to your role.  7. Write out your own personal, family emergency response plan using the template provided. Include enough detail that someone else looking at your plan would be able to follow it accurately. Share the plan with your family and everyone involved in carrying out your plan.  8. Draw a map showing how you would get from your home to your designated assignment location. Plan 3 different routes in case unexpected street closures obstruct your way.  9. Demonstrate use of communication equipment. See check-off list provided.  10. Indicate the ways in which you will be notified in the event of an emergency.  11. Attend an agency-sponsored lecture on How to Talk to People about Disasters.  12. Participate in a Communication Practice Session and practice the skills you learned during the lecture or make a presentation to others about how to talk to people about disasters. Describe the communication roles you have within the agency, with the media, with the general public, and with your family and personal contacts.  13. Participate in an agency led discussion to explore what kinds of unusual events or trends might indicate an emergency or disaster and describe appropriate actions.  14. Participate in an exercise/training in which you demonstrate creative problem solving to unusual challenges.  15. Participate in an agency-sponsored exercise to apply your knowledge of disaster preparedness to a real or made-up scenario and test your performance.  Additional competencies for public health leaders/ administrators   1. Describe the chain of command and management system for emergency response in jurisdiction 2. Communicate public health information/ role/legal authority accurately to all emergency response partners. 3. Maintain regular communication with emergency response partners 4. Ensure that agency has a written, updated plan for major categories of emergencies that respects the culture of community 5. Ensure that the agency regularly practices all parts of emergency response 6. Evaluate every emergency response/ drill to identify needed improvements 7. Ensure that knowledge/ skills gaps identifies through emergency response planning, drills and evaluation are filled   Additional for public health professionals   1. Demonstrate readiness to apply professional skills to a range of emergency situations during regular drills 2. Maintain regular communication with partner professionals in other agencies involved 3. Participate in continuing education to maintain up-to-date knowledge in area relevant to emergency response |

| Hites et al, 2007, USA [33] | Expand the existing national competency sets (by CDC/ Columbia University School of Nursing) and validate | All-hazard | Public health emergency responders | **Competency model and curriculum**   1. Develop emergency response communication network    1. Explain risk communication preparedness and planning, response, and recovery    2. List public health human resources essential to developing complete health risk communications preparedness and planning, response, and recovery efforts    3. Identify stakeholders, describe the process for developing stakeholder relationships, and summarize related emergency health risk communication strategies 2. Recognize and respond appropriately to psychological aspects of emergencies (eg, posttraumatic stress disorder prevention)    1. Describe the “psychology” of crisis and emergency health risk communication    2. Describe types of psychological effects caused by terrorist events    3. Describe psychological factors and effects in biological, chemical, and radiological threats 3. Describe the fundamentals of terrorism (eg, motives, tactics, types of terrorism)    1. List the motives of terrorism    2. List the tactics of terrorism    3. Identify reasons for terrorism 4. Recognize biological agents and resulting symptoms and respond appropriately to biological threats    1. Identify potential points of contamination for biological threats    2. Identify factors that affect dissemination of biological agents    3. Describe the range of consequences of a bioterrorist incident 5. Recognize chemical agents and resulting symptoms and respond appropriately to chemical threats    1. Identify multiple types of chemical agents used as food pathogens    2. Identify how different chemical interactions work    3. Define the scope of health impacts possible in a chemical terrorist event 6. Recognize radiological agents and resulting symptoms and respond appropriately to radiological threats    1. Explain the principles of ionizing radiation injury and attack management    2. List types of radiological weapons    3. Explain the effects of exposure to radiation 7. Recognize and employ effective counterterrorism techniques    1. List countermeasures to biological, chemical, and radiological terrorism    2. List some specialized response teams    3. Describe countermeasures to food borne attacks 8. Identify and assess health risks resulting from terrorist activities    1. Define basic health risk assessment and risk management concepts, and understand general health effects toxicology    2. Identify and explain the four steps of health risk assessment: hazard identification; dose-response assessment; exposure assessment, and risk characterization    3. Identify risk management issues 9. Identify abilities and limitations of other responders/agencies and use this knowledge in subsequent decision making    1. Distinguish myths from realities regarding people beyond the public health organization in relation to preparedness for bioterrorism, infectious disease outbreaks, and other public health threats and emergencies    2. Know, describe, and illustrate the best use of external Homeland Security agencies’ human resources for crisis health risk communication situations    3. Describe federal, state, and local responsibilities in responding to a terrorist incident 10. Apply safe work practices and avoid unnecessary risk     1. Identify and select appropriate PPE to use when approaching a potential biological, chemical, or radiological incident of unknown source     2. Identify and select appropriate PPE to use when handling chemical or biological terrorism agents. |
| --- | --- | --- | --- | --- |
| Tachivana T and Tachivana H, 2007, Japan [34] | Evaluate a competency-based training program (case method training) | All-hazard | Health emergency management agencies in the community | **Competency model and curriculum**  1. Estimation on switch from a peacetime system to an emergency system  2. Technical knowledge about medical and public health injury/damage spread (often at the same time as the initial investigation)  3. Power to perform prevention countermeasures against health injury/damage spread (often at the same time as the initial investigation)  4. Ability to collect information necessary for impact estimation  5. Power to perform the initial stage of an epidemiological investigation  6. Arrangement of coordination with and management ability of organizations engaged in technical investigations and surveys  7. Power to control the internal organization  8. Arrangement and coordination ability among outside organizations  9. Ability to set the targets for countermeasures and explain grounds for decisions inside and outside of one's own organization  10. Establishment of a system with clear responsibility and a simple decision-making process  11. Ability to promptly explain about necessary matters to victims, neighbouring residents, media or politicians, based on precise medical knowledge and a scientific viewpoint  12. To let others know about the lessons learnt from countermeasures from a positive perspective, not with a passive attitude  13. Actions for PTSD and to protect the most vulnerable in society  14. Power to achieve the realization of systematic improvements for residents after taking countermeasures  15. Ability to summarize a series of countermeasures in the form of reports and articles |

| Olson et. al, 2008, USA [35] | Delphi study with evaluation by 5256 surveys from participants | Bioterrorism and emergency | Public health workers | **Competency model and curriculum**  **Competencies**   1. Analytic assessment skills 2. Basic public health science skills 3. Cultural competency skills 4. Communication 5. Community dimensions of practice 6. Financial planning and management 7. Leadership and system thinking 8. Policy development and program planning skills   **Bioterrorism and emergency readiness curriculum plan**   1. Disaster preparedness (describe the public health role in emergency response, identify and locate the agency emergency response plan, identify limits to own knowledge, skill and authority and identify key system resource to solve) 2. Crisis/ risk communication (demonstrate correct use of all communication equipment used for emergency communication, describe communication role in emergency) 3. Incident command and management (describe the chain of command in emergency response, describe functional role in emergency response and demonstrate role in regular drills) 4. Surveillance (recognise unusual events that might indicate an emergency, appropriate action, apply creative problem-solving and flexible thinking, assessment of basic science) 5. Law (public health role in emergency response, public health policy) 6. Impact on community health (apply creative problem-solving and flexible thinking) 7. Understanding agents (recognise unusual event that might indicate an emergency, apply creative problem-solving and flexible thinking to unusual challenges) |
| --- | --- | --- | --- | --- |
| Olu et al., 2018, Rwanda [41] | Mixed methods study | All-hazard | African health workforce in public health disaster risk management | **Competency model and curriculum**  **14 Health DRM Core Competencies and Course Content of Basic and Intermediate Course**  1. Demonstrate knowledge of public health principles and practices for Disaster Risk Management   - Disaster risk management concepts - Public health consequences of disasters - Context: political, social and economic environment   2. Demonstrate knowledge of basic epidemiological methods and data management   - Basic epidemiology - Data analysis and management   3. Demonstrate the ability to communicate effectively in DRM   - Key principles - Risk communication - Operational communication (any communication that is not with the media and public)   4. Demonstrate the knowledge of principles of legal, human rights and ethics in dealing with DRM   - Ethics - Human rights - International humanitarian law - International health regulations   5. Demonstrate ability to identify, mobilise and manage resources   - Resource mobilization   6. Demonstrate the ability to apply logistics management   - Logistics management   7. Demonstrate the ability to apply measures of safety and security   - Basic security in the field - Protection and family safety   8. Demonstrate effective leadership, teamwork and management skills required for DRM   - Principles of leadership, management and coordination - Leadership - Management - Coordination   9. Demonstration knowledge about the monitoring and evaluation cycle   - Key principles - Monitoring - Evaluation   10. Demonstrate the ability to conduct capacity assessments   - Key principles - Risk assessments - Needs assessments   11. Demonstrate the ability to plan and implement preventive and mitigation activities   - Key principles - Risk reduction - Mitigation   12. Demonstrates the ability to plan and implement emergency preparedness at community and health facility levels   - Key principles - Planning - Early warning - Surge capacity - Training - Exercise management   13. Demonstrate ability to apply DRM principles and practices for the health response to disasters and public health emergencies   - Key principles - Health assessment - MCM /EMS - Incident management - Public health programmes in emergencies - Planning   14. Demonstrate ability to plan and implement health system and population recovery.   - Key principle - Recovery needs assessments - Recovery strategy and planning - Programme implementation   Other training   - Integrated Simulation |
| Ripoll-Gallardo et al, 2020, Italy [43] | Training and pilot evaluation study by MSF-Italy and CRIMEDIM | Humanitarian field projects, including armed conflict areas or following natural or man-made disasters | Senior residents (doctors) in anesthesia & critical care, emergency medicine, and paediatrics | **Competency model and curriculum**  Disaster medicine   - Understand the definition and different phases of disasters. - Define the nature of injury or illness in relation to different types of disasters. - Describe objectives and features of disaster medicine. - Understand the international disaster response mechanism with involved bodies and organizations.   Incident Management System (IMS)   - Describe the general principles and different phases of the IMS. - Demonstrate ability to work within an IMS. - Describe the concept and different methods of Mass Casualty Triage. - Define the concept of surge capacity and its role in unforeseen emergencies and disasters.   Communication   - Recognize a disaster in progress, assess and report the situation. - Define and apply the principles of successful communication with local and expatriate staff, within and among organizations and with the media during emergencies. - Describe the radio communication procedures and protocols. - Recognize the importance of post-event reports.   Resource management   - Manage supplies, drugs and equipment and other resources for an effective response. - Manage, supervise, and appropriately use local staff and expatriate aid workers during emergencies.   Public health   - Recognize the top priorities for public health interventions during complex emergencies. - Describe indicators used to assess and monitor public health during complex emergencies. - Understand key epidemiological principles and terminology. - Define the minimum levels to be attained in humanitarian interventions regarding the provision of water, sanitation and hygiene. - Define the minimum levels to be attained in humanitarian interventions regarding the provision of food and nutrition. - Identify which infectious diseases can constitute a major threat following a disaster according to the geographical location and the type of emergency occurring.   Safety and security   - Understand the need for a safe and secure approach in humanitarian environments. - Analyze the security environment on the basis of the seven pillars of security. - Apply the preventive measures and/or individual or collective responsibilities adapted to each form of stress. - Identify sources of risk, describe risk scenarios and identify risk mitigation measures.   Ethics and international humanitarian law   - Apply basic principles of medical ethics to disaster situations. - Recognize and react accordingly to the difficulties entailed by humanitarian scenarios where different cultural backgrounds are represented. - Define the concept and understand the origins of International Humanitarian Law - List the main International Human Rights - Describe the role of International Humanitarian Law in in protecting the dignity and rights of the most vulnerable populations during armed conflicts   Situational awareness   - Respond appropriately to an ever-changing environment and stress-induced situations. - Adapt to pressure and change to operate effectively within humanitarian contexts.   Psychological support   - Describe the main psychological needs in emergency contexts. - Describe the essential criteria to organize actions in psychological support. - Apply the principles of psychological first aid in emergency situations   Leadership   - Understand the definition of leadership and recognize the importance in an emergency context. - Describe the different management styles. - Understand conflict management and modify one’s own management style. - Apply the principles of Non-Violent communication.   Clinical considerations in the specific field of Anesthesia, Pediatrics and Emergency Medicine in Low Resource Settings  Understand and apply the principles of safe anesthesia, emergency medicine or pediatrics in low-resource settings according to the needs and resource available. |

Supplementary 3b: Summary table for competency models identified in English and Japanese language literature review

| Author, Year, Country | Type of article | Hazard | Target sample | Content |
| --- | --- | --- | --- | --- |
| Hsu et al, 2006, USA [32] | Expert panel review | All-hazards | Health professionals in disaster | **Competency model**  7 cross-cutting competencies  1. Recognize a potential critical event and implement initial actions  2. Apply the principles of critical event management  3. Demonstrate critical event safety principles  4. Understand the institutional emergency operations plan  5. Demonstrate effective critical event communications  6. Understand the incident command system and your role in it  7. Demonstrate the knowledge and skills needed to fulfill your role during a critical event |
| Subbarao et al, 2008, USA [36] | Expert panel conducted systematic review and developed a new educational framework by Delphi method | All-hazard | All health professionals in disaster and specifically for 3 categories (informed workers/ students, practitioners, leader) | **Competency model**  1. Preparation and Planning  1.1 Demonstrate proficiency in the use of an all-hazards framework for disaster planning and mitigation.  1.2 Demonstrate proficiency in addressing the health-related needs, values, and perspectives of all ages and populations in regional, community, and institutional disaster plans.  2. Detection and Communication  2.1 Demonstrate proficiency in the detection of and immediate response to a disaster or public health emergency.  2.2 Demonstrate proficiency in the use of information and communication systems in a disaster or public health emergency.  2.3 Demonstrate proficiency in addressing cultural, ethnic, religious, linguistic, socioeconomic, and special health-related needs of all ages and populations in regional, community, and institutional emergency communication systems.  3. Incident Management and Support Systems  3.1 Demonstrate proficiency in the initiation, deployment, and coordination of national, regional, state, local, and institutional incident command and emergency operations systems.  3.2 Demonstrate proficiency in the mobilization and coordination of disaster support services.  3.3 Demonstrate proficiency in the provision of health system surge capacity for the management of mass casualties in a disaster or public health emergency.  4. Safety and Security  4.1 Demonstrate proficiency in the prevention and mitigation of health, safety, and security risks to yourself and others in a disaster or public health emergency.  4.2 Demonstrate proficiency in the selection and use of personal protective equipment at a disaster scene or receiving facility.  4.3 Demonstrate proficiency in victim decontamination at a disaster scene or receiving facility.  5. Clinical/Public Health Assessment and Intervention  5.1 Demonstrate proficiency in the use of triage systems in a disaster or public health emergency.  5.2 Demonstrate proficiency in the clinical assessment and management of injuries, illnesses, and mental health conditions manifested by all ages and populations in a disaster or public health emergency.  5.3 Demonstrate proficiency in the management of mass fatalities in a disaster or public health emergency.  5.4 Demonstrate proficiency in public health interventions to protect the health of all ages, populations, and communities affected by a disaster or public health emergency.  6. Contingency, Continuity, and Recovery  6.1 Demonstrate proficiency in the application of contingency interventions for all ages, populations, institutions, and communities affected by a disaster or public health emergency.  6.2 Demonstrate proficiency in the application of recovery solutions for all ages, populations, institutions, and communities affected by a disaster or public health emergency.  7. Public Health Law and Ethics Knowledge and skills  7.1 Demonstrate proficiency in the application of moral and ethical principles and policies for ensuring access to and availability of health services for all ages, populations, and communities affected by a disaster or public health emergency.  7.2 Demonstrate proficiency in the application of laws and regulations to protect the health and safety of all ages, populations, and communities affected by a disaster or public health emergency. |
| Tachivana et al., 2011, Japan [38] | Delphi study on  participants working at the department of health emergency response at public health sectors | All-hazard | Public health workers | **Competency model**  1. Ability to estimate the impact on community health from the first report or the initial investigation  2. Management ability of investigation of causes  3. Organizational management ability to implement countermeasures  4. Ability to provide prompt and accurate information and to promptly explain of facts found, countermeasures, and policies. Role as a spokesperson.  5. Ability to establish a system with sustainable recurrence prevention measure and to create social consensus  6. Competencies required in ordinary time |
| Walsh et al, 2012, USA [39] | Competency set reviewed by expert panel and survey | All-hazard | Health professionals in disaster medicine and public health | **Competency model**  **11 core competencies and 36 sub-competencies**  1. Demonstrate personal and family preparedness for disasters and public health emergencies  1.1 Prepare a personal/family disaster plan  1.2 Gather disaster supplies/equipment consistent with personal/family plan  1.3 Practice one’s personal/family disaster plan annually  1.4 Describe methods for enhancing personal resilience, including physical and mental health and well-being, as part of disaster preparation and planning  2. Demonstrate knowledge of one’s expected role(s) in organizational and community response plans activated during a disaster or public health emergency  2.1 Explain one’s role within the incident management hierarchy and chain of command established within one’s organization/agency in a disaster or public health emergency  2.2 Prepare a personal professional disaster plan consistent with one’s overall agency, organizational, and/or jurisdictional plan  2.3 Explain mechanisms for reporting actual and potential health threats through the chain of command/authority established in a disaster or public health emergency  2.4 Practice one’s personal professional disaster plan in regular exercises and drills  3. Demonstrate situational awareness of actual/potential health hazards before, during, and after a disaster or public health emergency  3.1. Identify general indicators and epidemiological clues that may signal the onset or exacerbation of a disaster or public health emergency  3.2. Describe measures to maintain situational awareness before, during, and after a disaster or public health emergency  4. Communicate effectively with others in a disaster or public health emergency  4.1. Identify authoritative sources for information in a disaster or public health emergency  4.2. Explain principles of crisis and emergency risk communication to meet the needs of all ages and populations in a disaster or public health emergency  4.3. Identify strategies for appropriate sharing of information in a disaster or public health emergency  4.4. Identify cultural issues and challenges in the development and dissemination of risk communication in a disaster or public health emergency  5. Demonstrate knowledge of personal safety measures that can be implemented in a disaster or public health emergency Explain general health, safety, and security risks associated with disasters and public health emergencies  5.1. Describe risk reduction measures that can be implemented to mitigate or prevent hazardous exposures in a disaster or public health emergency  6. Demonstrate knowledge of surge capacity assets, consistent with one’s role in organizational, agency, and/or community response plans  6.1. Describe the potential impact of a mass casualty incident on access to and availability of clinical and public health resources in a disaster or public health emergency  6.2. Identify existing surge capacity assets which could be deployed in a disaster or public health emergency  7. Demonstrate knowledge of principles and practices for the clinical management of all ages and populations affected by disasters and public health emergencies, in accordance with professional scope of practice  7.1 Discuss common physical and mental health consequences for all ages and populations affected by a disaster or public health emergency  7.2 Explain the role of triage as a basis for prioritizing or rationing health care services for all ages and populations affected by a disaster or public health emergency  7.3 Discuss basic lifesaving and support principles and procedures that can be utilized at a disaster scene  8. Demonstrate knowledge of public health principles and practices for the management of all ages and populations affected by disasters and public health emergencies  8.1 Discuss public health consequences frequently seen in disasters and public health emergencies  8.2 Identify all ages and populations with functional and access needs who may be more vulnerable to adverse health effects in a disaster or public health emergency  8.3 Identify strategies to address functional and access needs to mitigate adverse health effects of disasters and public health emergencies  8.4 Describe common public health interventions to protect the health of all ages and populations affected by a disaster or public health emergency  9. Demonstrate knowledge of ethical principles to protect the health and safety of all ages, populations, and communities affected by a disaster or public health emergency  9.1 Discuss ethical issues likely to be encountered in disasters and public health emergencies  9.2 Describe ethical issues and challenges associated with crisis standards of care in a disaster or public health emergency  9.3 Describe ethical issues and challenges associated with allocation of scarce resources implemented in a disaster or public health emergency  10. Demonstrate knowledge of legal principles to protect the health and safety of all ages, populations, and communities affected by a disaster or public health emergency  10.1 Describe legal and regulatory issues likely to be encountered in disasters and public health emergencies  10.2 Describe legal issues and challenges associated with crisis standards of care in a disaster or public health emergency  10.3 Describe legal issues and challenges associated with allocation of scarce resources implemented in a disaster or public health emergency  10.4 Describe legal statutes related to health care delivery that may be activated or modified under a state or federal declaration of disaster or public health emergency  11. Demonstrate knowledge of short- and long-term considerations for recovery of all ages, populations, and communities affected by a disaster or public health emergency  11.1 Describe clinical considerations for the recovery of all ages and populations affected by a disaster or public health emergency  11.2 Discuss public health considerations for the recovery of all ages and populations affected by a disaster or public health emergency  11.3 Identify strategies for increasing the resilience of individuals and communities affected by a disaster or public health emergency  11.4 Discuss the importance of monitoring the mental and physical health impacts of disasters and public health emergencies on responders and their families |
| Schultz et al, 2012, USA [40] | Delphi method | All-hazard | Acute care medical professionals (ED nurse, physicians, out-of-hospital EMS) | **Competency model**  **19 core competencies (93 objectives)**  1. Comprehend orders, tasks, requests, conversations and other forms of communication, including professional vocabulary, related to all-hazard preparedness and response  2. Demonstrate an ability to follow and work within an IMS  3. Recognize a disaster is in progress, assess and report the situation, initiate the disaster plan, notify the appropriate persons/ agencies, and identify important data for inclusion in post-event report  4. Communicate effectively and efficiently within and among agencies, as well as with the media, during any disaster  5. Manage supplies, pharmaceuticals, equipment, and other resources for an effective response  6. Manage, supervise, and appropriately use volunteers  7. Use the resources provided by GOs and NGOs effectively (such as DMAT, USAR, MRC, and the Red Cross)  8. Prevent and mitigate risks to self and others  9. Prioritize patients to maximize survivability  10. Participate in a process that secures adequate personnel, supplies, equipment, and space for patient care  11. Use recordkeeping processes to ensure continuity of patient information  12. Facilitate or perform patient transport effectively and safely during a disaster  13. Decontaminate patients or staff, following appropriate procedures  14. Manage patients with presentations that commonly occur during specific types of disasters, eg, environmental illnesses; burns; blast and crush injuries; nuclear, biologic, and chemical exposure  15. Manage patients within each special-needs population, as appropriate, according to their specific psychosocial, medical, cultural, age, and logistic need  16. Perform evacuation, as needed, using pre- event evacuation plans and maintaining essential medical information with each patient  17. Synthesize information and formulate new plans in an ever- changing environment  18. Apply basic principles of medical ethics to disaster situations  19. Respond appropriately to stress- induced and other behaviours in patients, responders, and others during a disaster |
| The International Federation of Red Cross and Red Crescent Societies (IFRC), 2019, International [42] | Core Competency Framework produced by IFRC | All-hazard | All surge personnel including volunteers (not health specific) | **Core Competency Framework for Surge Personnel**  **20 Competencies, Each Having 4 Levels (Foundational Tier, Tier 1, Tier 2, Tier 3**  Red Cross Red Crescent competencies   1. Movement context, principles and values 2. National Society Capacity Strengthening   Operational competencies   1. Coordination 2. Assessment 3. Direction Setting and Quality Programme Management 4. Information Management 5. Resource Management 6. Safety and Security 7. Transition and Recovery   Cross-Cutting competencies   1. Community engagement and accountability 2. Protection, Gender and Inclusion 3. Environmental Sustainability   Behavioural competencies   1. Collaboration and Teamwork 2. Conflict Management 3. Interpersonal Communication 4. Cultural Awareness 5. Judgement and Decision Making 6. Motivating Others 7. Personal Resilience 8. Integrity |

Supplementary 3c: Summary table for curricula (without a competency model) identified in English and Japanese language literature review

| Author, Year, Country | Type of article | Hazard | Target sample | Content |
| --- | --- | --- | --- | --- |
| Rottman et al, 2005, USA [30] | Describe the development of 2-day training curriculum in emergency public health. Outcome was measured after training | Natural disasters and bioterrorism | Public health professionals | **Training curriculum**  Topic agenda for 2-day competency-based training in Emergency Public Health (contents are matched to the 9 core competencies in emergency preparedness – CDC/ Columbia University)  Day 1   - Impact of natural disasters on public health - Table top scenario: natural hazard - Multiuse emergency management planning - Major public health functions in disasters - *Content:* A review of the role and function of public health professionals and agencies during a disaster - Legal authority and responsibility of the public health officer - Standardized emergency management system and operational areas - *Content*: Define NIMS and the use of operational areas to communicate and coordinate during emergency events - Public health incident command system - *Content*: Describe the incident command system, including roles of individuals within the incident command system structure - Table top scenario: naturally occurring disease outbreak   Day 2   - Disease outbreak versus natural disasters - Bioterrorism 101 - *Content*: Provide an overview of Category A agents, the clinical recognition of those agents, and public health response options for events involving these agents, and public health response options for events involving these agents - Scenario exercise: chemical/ radiological terrorism event - Bioterrorism versus other outbreaks - Public health’s role in bioterrorism - Integration of public health into local emergency management - Coordination with hospitals and healthcare providers - Scenario exercise: bioterrorism event - *Content*: Respond to an event that challenges interagency management, risk communication, collaboration with federal agencies, planning for mass illness/ vaccination programs, and assessment of effectiveness of public health response |
| Calhoun et al, 2005, USA [31] | Integrate competency-based learning and assessment in education initiatives | All-hazard | Public health workers | **Training curriculum**  Skill stages that the particular course to be targeted in the curriculum (CDC competency) planning   - Awareness stage (new learner) - Know about the key task associated with a communication role - Comprehend the importance of communication role - Proficiency stage (advanced learner) - Effectively and efficiently use established communications systems within an agency - Mastery stage (highly experienced learner) - Develop a plan for conducting training exercises for emergency response communication simulation activities - Evaluate a trainee reporting an emergency to the media |
| Coule et. al, 2009, USA [37] | Delphi method with evaluation by participants | All-hazard | Health care professionals and Emergency response personnel | **Training curriculum**   1. Core Disaster Life Support (Internet-based)  - Focused on medical aspects of first responder or above  1. Basic Disaster Life Support (8-hour didactic program)  - for those at EMT/ paramedic level or above - Content - DISASTER Paradigm and All-Hazards Preparedness - Mass Triage - Natural Disasters - Traumatic and Explosive Events - Nuclear and Radiological Events - Chemical Events - Biological Events (including naturally occurring infectious diseases) - Public Health and Local Disaster Response - Course Evaluation and Testing  1. Advanced Disaster Life Support (16-hour didactic)  - for medical first responder and paramedic level and above - Content - Review - Triage Exercise - Mass Fatality Management - Medical Decontamination - Community Disaster Planning - Small Group Exercises - Clinical Patient Exercises - MASS Triage Exercise - Decontamination Exercise - Disaster Clinical Skills (Mass Immunization and Mass prophylaxis) - Course Evaluation  1. National Disaster Life Support Decontamination (12-hour)  - for hospital-based personnel on decontamination - Content - Event recognition - Personal protective equipment - Ambulatory decontamination - Litter decontamination - Surveying for radiological materials - Full-scale decontamination exercise |

Supplementary 3d: Gap analysis of published competency model and curricula against the WHO Health Emergency Programme Core Competencies and health EDRM perspectives

| Author, Year, Country | Competency model or curricula | Change | Communication | Teamwork | Partnerships | Leadership | Technical emergency preparedness | Technical emergency response | Technical emergency recovery | Comprehensive emergency management perspectives | Risk-based approach |
| --- | --- | --- | --- | --- | --- | --- | --- | --- | --- | --- | --- |
| Columbia University/CDC, 2002-2005, USA [25-29] | Both |  | x |  |  |  | x | x |  |  |  |
| Hites et al, 2007, USA [33] | Both |  | x |  | x |  | x | x | x | x | x |
| Tachivana T and Tachivana H, 2007, Japan [34] | Both |  | x |  | x |  | x | x |  |  |  |
| Olson et. al, 2008, USA [35] | Both |  | x |  |  | x | x | x |  |  |  |
| Olu et al., 2018, Rwanda [41] | Both |  | x |  | x | x | x | x | x | x | x |
| Ripoll-Gallardo et al, 2020, Italy [43] | Both |  | x |  |  | x |  | x |  |  |  |
| Hsu et al, 2006, USA [32] | Competency model only |  | x |  |  |  | x | x |  |  |  |
| Subbarao et al, 2008, USA [36] | Competency model only |  | x |  |  |  | x | x | x |  |  |
| Tachivana et al., 2011, Japan [38] | Competency model only |  | x |  | x |  |  | x |  |  |  |
| Walsh et al, 2012, USA [39] | Competency model only |  | x |  | x |  | x | x | x |  | x |
| Schultz et al, 2012, USA [40] | Competency model only |  | x |  | x |  | x | x |  |  |  |
| IFRC, 2018 [42] | Competency model only |  | x | x | x |  |  | x | x |  |  |
| Rottman et al, 2005, USA [30] | Curriculum only |  | x |  | x |  | x | x |  |  |  |
| Calhoun et al, 2005, USA [31] | Curriculum only |  | x |  |  |  |  |  |  |  |  |
| Coule et. al, 2009, USA [37] | Curriculum only |  |  |  | x |  | x | x |  |  |  |

Supplementary 3e: Gap analysis of survey competency model against selected WHO Health Emergency Programme Core Competencies and health EDRM perspectives


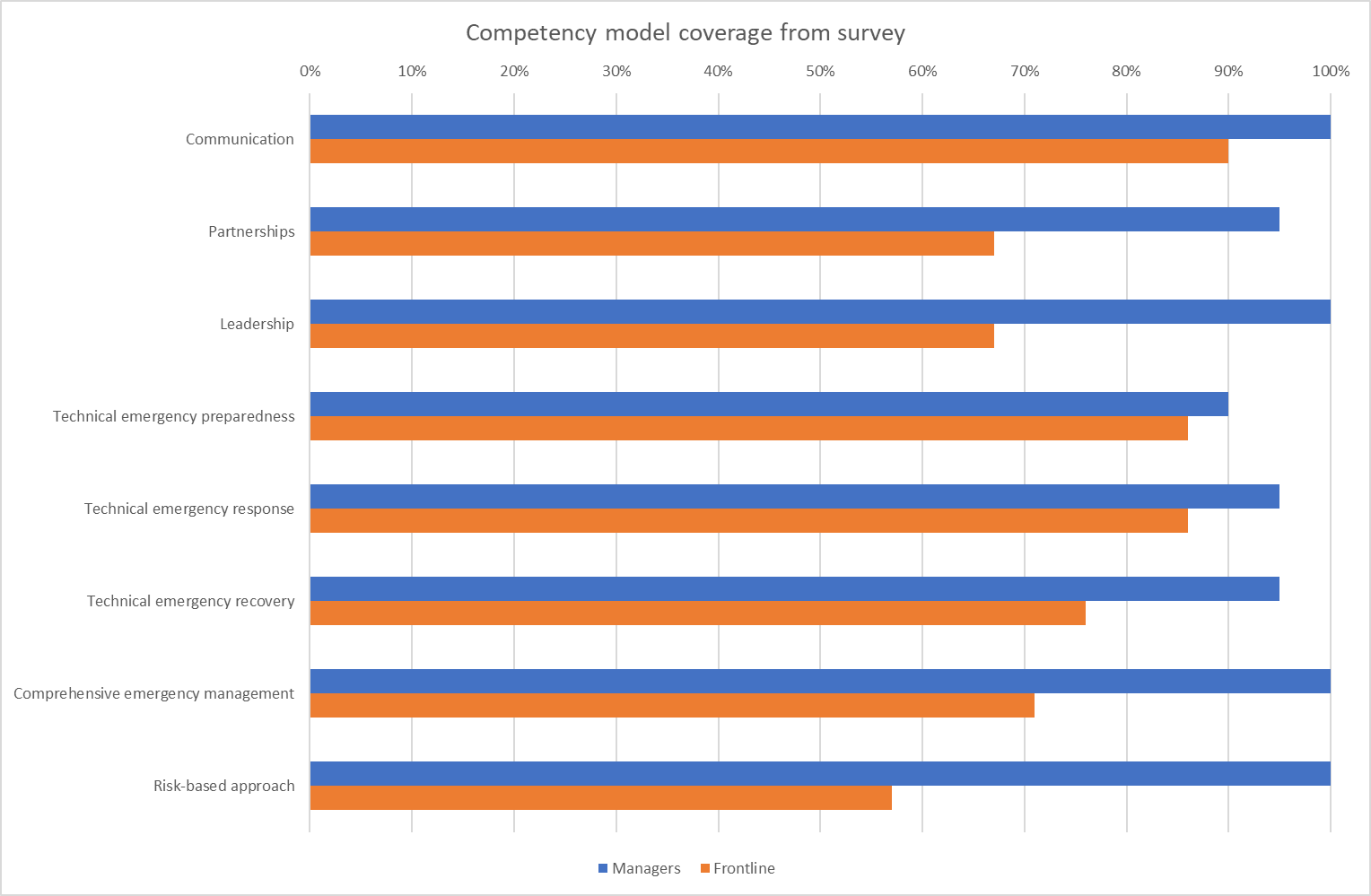


Supplementary 3f: Gap analysis of survey curricula against selected WHO Health Emergency Programme Core Competencies and health EDRM perspectives


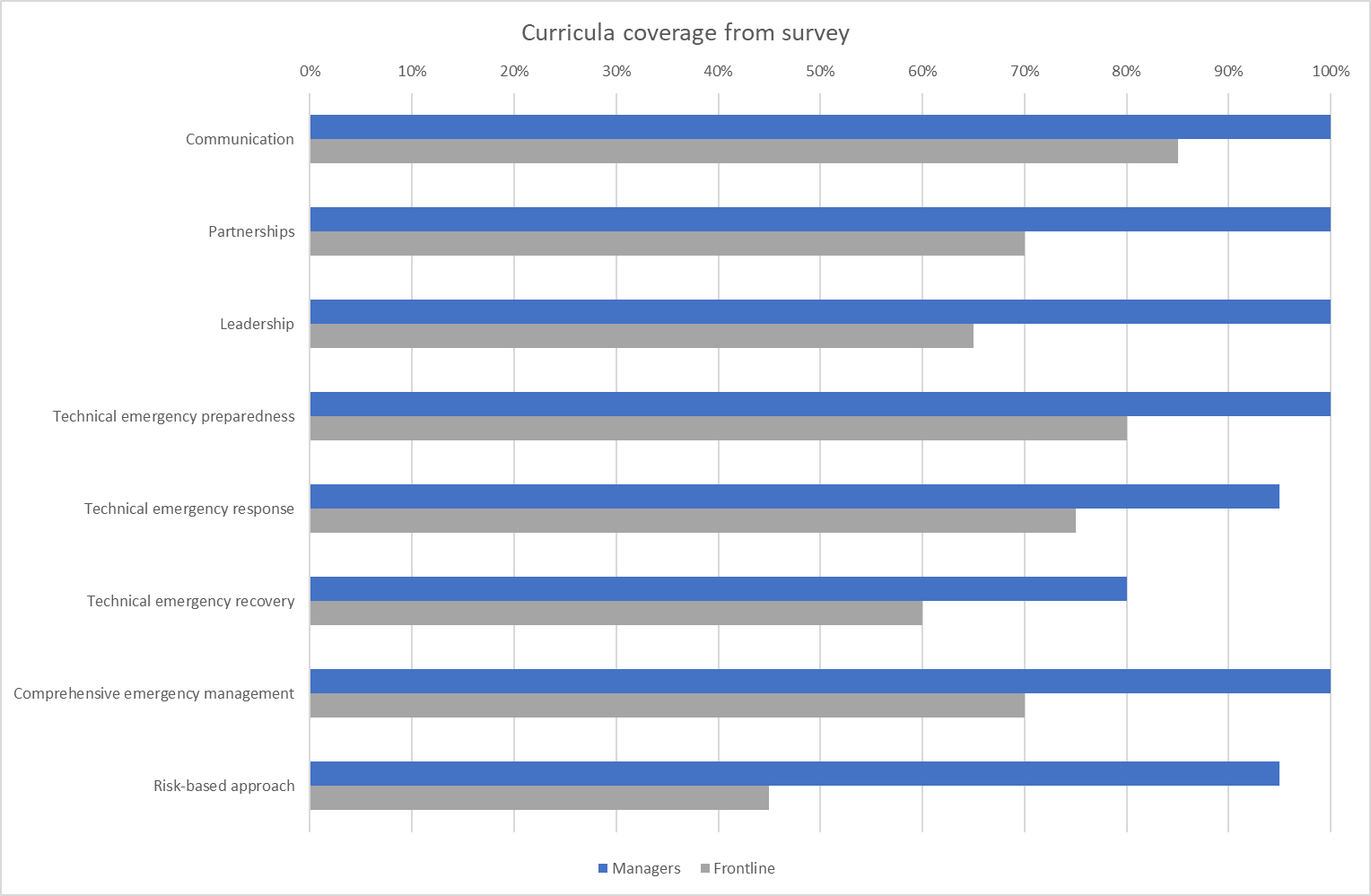


Supplementary 3g: Assessment of competency attainment in published curricula with a competency model

| Author, Year, Country | Assessment method |
| --- | --- |
| Columbia University/CDC, 2002-2005, USA [25-29] | Take Columbia online course or equivalent  1. Pre-test/Post-test scores  2. Columbia evaluation form (online)  Participate in a training about Weapons of Mass Destruction—Online or Agency Sponsored  1. Pre-test/Post-test scores  Participate in role-specific training  1. Pre-test/Post-test scores  2. Student evaluation of trainings score  3. Exercise performance self-assessment scores  Participation in planning and implementation of worksite plan to ensure safety/security in event of emergency  1. Scores on “pop quizzes”  2. Exercise Performances self-assessment scores  Identify place on incident command chart  1, 2. Pre-test/Post-test scores  1, 2. Scores on “Pop quizzes”  Identify and locate PHS emergency response plan  1. Pre-test/Post-test scores  1. Scores on “pop quizzes”  2. Employee assessment of accessibility of response plan  Attend an agency-sponsored lecture on  “Personal and Family Emergency Preparedness”; then write out personal family response plan  1. Pre-test/Post-test scores  2. Compatibility with Red Cross family preparedness checklist  Draw map from home to emergency assignment location  1. Scores on “Pop quizzes”  Indicate ways in which you will be notified in event of an emergency  1. Scores on “pop quizzes”  Attend an agency sponsored lecture on How  to Talk to People about Disasters  1. Pre-test/post-test scores  1. Scores on “pop quizzes”  2. Student evaluation of trainings  3. Exercise performance self-assessment scores  Participate in a Communication Practice Session; describe your emergency communication roles  1. Pre-test/Post-test scores  1. Scores on “pop quizzes”  2. Student evaluation of trainings  3. Exercise performance self-assessment scores  Participate in agency-led discussion on emergency recognition and describe appropriate actions  1. Pre-test/Post-test scores  a. Before and after agency-led discussion on emergency recognition/ (Short Term)  b. Repeat “post-test” annually at one, two, and three year intervals following initial training/(Long Term)  1. Scores on “pop quizzes”  a. At three month intervals X 3 years following completion of agency-led discussion on emergency recognition (Short and Long Term)  2. Exercise performance scores (self-assessment)/(Short Term)  a. Upon completion of exercise/(Short Term)  Participate in an exercise/ training in which you demonstrate creative problem solving  1. Training Evaluations  a. Upon completion of exercise training/ (Short Term)  2. Exercise Performance Results (self-assessment)  a. Upon completion of exercise/(Short Term)  Participate in an agency-sponsored exercise to apply your knowledge of disaster preparedness to a real or made-up scenario and test your performance.  1. Exercise Performance Results (self-assessment)  a. Upon completion of exercise/(Short Term) |
| Hites et al, 2007, USA [33] | Though many goals were listed, the method of assessing the participants was not specified. |
| Tachivana T and Tachivana H, 2007, Japan [34] | Self-assessment of attainment of competencies were used. |
| Olson et. al, 2008, USA [35] | The hybrid Kirkpatrick model was used as a mean of assessing how effective the curriculum was in the form of the “Disaster in Franklin County” gaming simulation. However, the exact finding of the evaluation is not included in the publication. |
| Olu et al., 2018, Rwanda [41] | Their assessment methods differ depending on the different levels of competence (basic, intermediate and advanced). They also include feedback from the participants.  Basic Training in Health Disaster Risk Management  - Continuous assessments: learning will be measured through observation, written tasks (at the end of each session, each day, one week),  - Final assessments: through tests (essay, pre and post-test)  Intermediate Training in Health Disaster Risk Management  - Continuous assessments: Learning will be measured through observation, written tasks (at the end of each session, each day, one week),  - Final assessments: through tests (essay, pre and post-test)  - The course evaluation will be done by the participants at the end of the training and recommendation made.  Advanced Training in Health Disaster Risk Management  - Observation and recommendation of the field supervisors (20%)  - Written field report (50%)  - Verbal report – defence of the report (30%).  - The course evaluation will be done by the participants at the end of the training and recommendations made. |
| Ripoll-Gallardo et al, 2020, Italy [43] | Feedback is given the participants using a 5-point Likert scale questionnaire with a separate space for commentaries and personal opinions.  Competencies are assessed in the following manner:  - Knowledge with a 30-question-multiple-choice test.  - Attitude with a 12-question-5-point Likert scale questionnaire. In this study, the term “attitude” was defined as the students’ positive or negative predisposition toward the competency domains at the basis of the course.  - Skills with simulation-based performance tests, in which each student acted as lead physician in the management of a critically-ill patient in a low-resource emergency room. |

Supplementary 3h: Knowledge and skills listed by survey respondents not included in the list of management and technical competencies

| Competency models:  Mass casualty management, Mass fatality management, Research for Health EDRM, Ethics, Climate change, Mental health and psychosocial support, Continuity of health services/essential health functions in emergencies, understanding resilience, Humanitarian principles  Curricula:  Courses on ethics; Prevention of sexual exploitation and abuse; Human rights/gender/disability; Epidemic preparedness and response, One Health, Climate change adaptation; After action reviews; Health EDRM research (underway); Specific diseases (e.g. Ebola, COVID-19), Courses for infectious disease management; courses for management of chemical safety and incidents; courses for radiological protection and emergency management; nutrition in emergencies; mental health and psychosocial support; infodemic management |
| --- |
